# Supplementary material for: Werner syndrome exonuclease promotes gut regeneration and causes age-associated gut hyperplasia in Drosophila
Source: PLoS Biol. 2025 Apr 22;23(4):e3003121. doi: 10.1371/journal.pbio.3003121 (PMC12013949; doi:10.1371/journal.pbio.3003121)
Supplement: S1 Fig — Underlying data and statistical analysis in S1 Data. (DOCX) [file pbio.3003121.s001.docx]

**
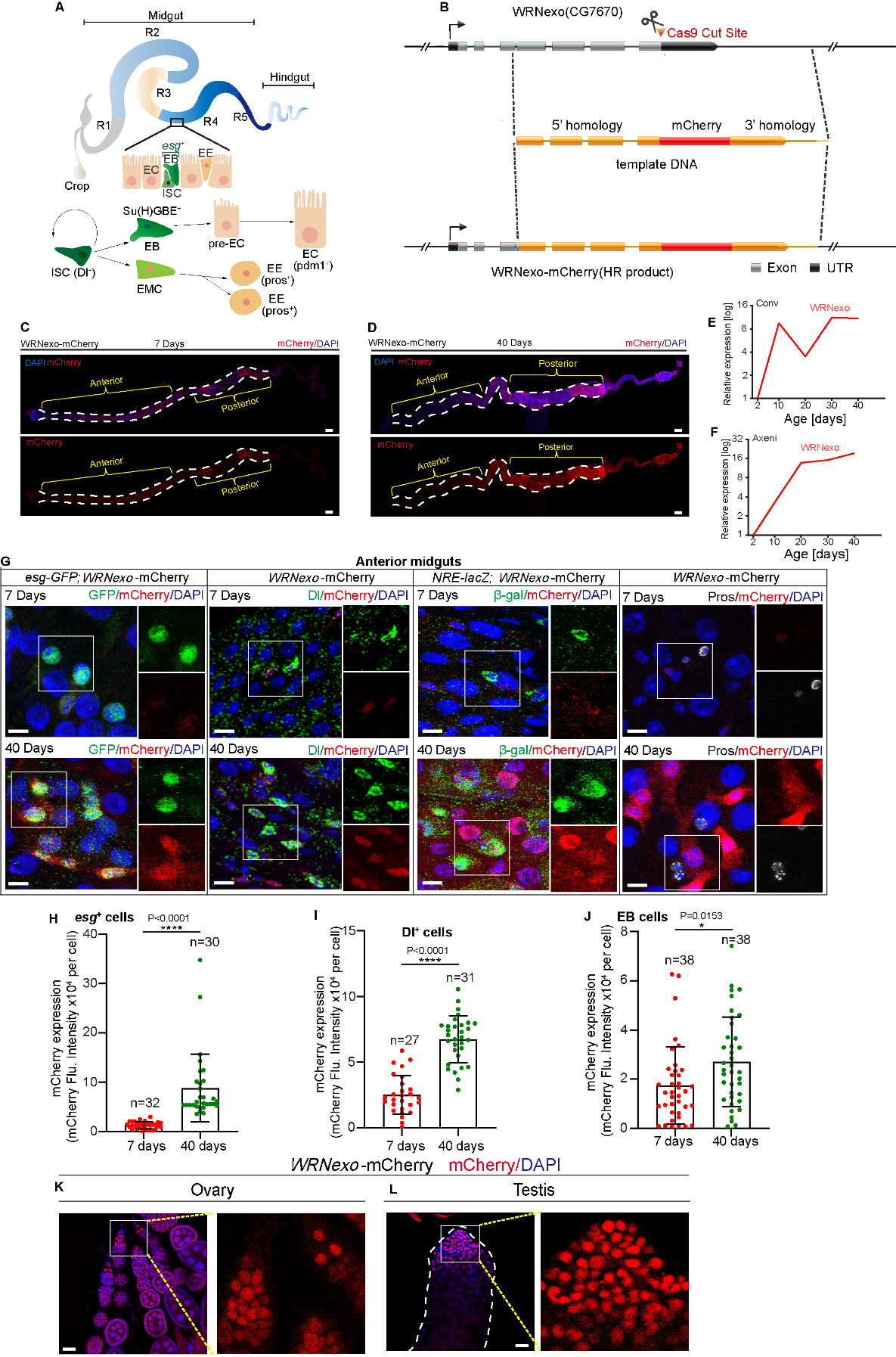
**

**S1 Fig. WRNexo is expressed in *Drosophila* midguts and the expression increases with aging,** **related to Fig 1.**

(A) Cartoon indicating five regions (R: region) and a model of different cell types in the adult midgut. An ISC (Dl^+^ and *esg*^+^) divides asymmetrically once to generate a new ISC as well as a diploid precursor enteroblast (EB; *esg*^+^ and NRE^+^) or a diploid precursor enteroendocrine mother cell (EMC; *esg*^+^ and Pros^+^). The post-mitotic EB differentiates further into tetraploid premature enterocytes (pre-ECs) (*esg*^+^ and Pdm1^+^), which then differentiate into octoploid mature ECs (Pdm1^+^). The EMC divides once to form two diploid enteroendocrine cells (EEs; Pros^+^).

(B) Model of the strategy used to construct the endogenous *WRNexo-mCherry* knock-in *Drosophila* line. mCherry sequence was added to the end of the WRNexo open reading frame (ORF) and the WRNexo-mCherry fusion protein was obtained.

(C, D) Representative images of whole midguts with the endogenous *WRNexo-mCherry* line in young (C) and old flies (D). Single channel of mCherry is shown in the lower panel.

(E, F) Relative expression trends of the WRNexo gene in midguts over time in flies cultured under conventional (Conv, E) and axenic (Axeni, F) conditions.

(G) Immunofluorescence images of anterior midguts of young (upper panels) and old flies (lower panels) with different cell markers staining for WRNexo-mCherry. Similar to posterior midguts, all of WRNexo-mCherry^+^ cells expressed *esg*-GFP; WRNexo-mCherry expressed in ISCs (Dl^+^ cells) and WRNexo-mCherry expressed in EBs (NRE^+^ cells). WRNexo-mCherry was not expressed in EEs (Pros^+^ cells).

(H, I, J) Quantification of mCherry fluorescence intensity in *esg*-GFP^+^ (H), Dl^+^ (I), and NRE^+^ (J) cells from young and aged anterior midguts. Each dot represents one cell.

(K, L) Representative images of ovaries (K) and testes (L) with the endogenous *WRNexo-mCherry* reporter line. The boxed areas in (K, L) are enlarged to the right with a single channel.

DAPI-stained nuclei (blue). Scale bars represent 200 μm in C, D, 10 μm in K, L, 5 μm in G. Error bars represent SD. Student’s t-tests, **p* < 0.05, ***p* < 0.01, ****p* < 0.001, *****p* < 0.0001, and NS (non-significant) represents *p* > 0.05. Underlying data and statistical analysis in S1 Data.
