# Supplementary material for: Werner syndrome exonuclease promotes gut regeneration and causes age-associated gut hyperplasia in Drosophila
Source: PLoS Biol. 2025 Apr 22;23(4):e3003121. doi: 10.1371/journal.pbio.3003121 (PMC12013949; doi:10.1371/journal.pbio.3003121)
Supplement: S2 Fig — Underlying data and statistical analysis in S2 Data. (DOCX) [file pbio.3003121.s002.docx]

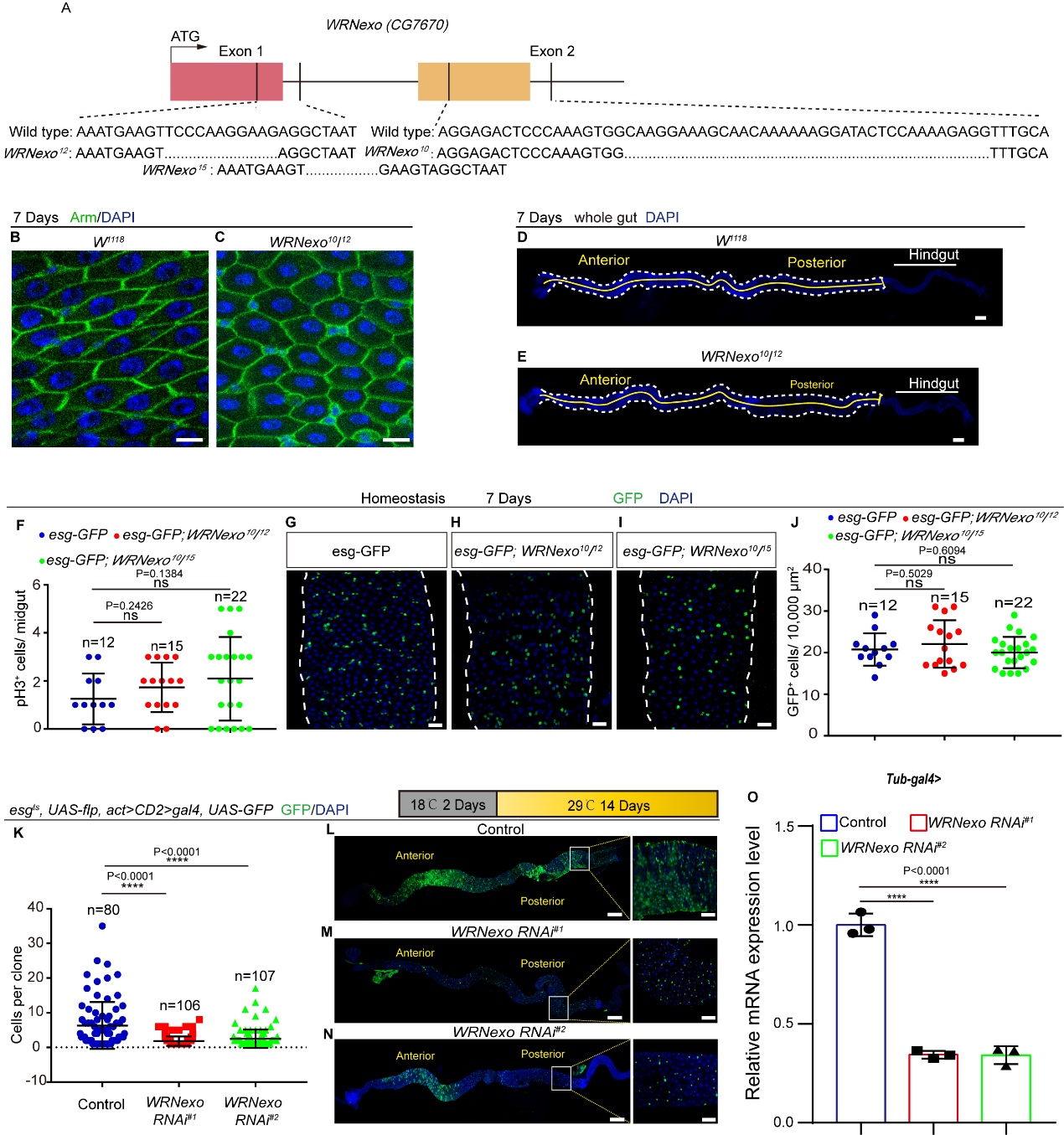


**S2 Fig. WRNexo is essential for gut homeostasis, related to Fig 2.**

(A) Schematic summary of *WRNexo* mutant alleles generated by the CRISPR-Cas9

method.

(B, C) Representative images after DAPI and Armadillo staining of young (7 days) flies. There was no significant difference in expression between epithelial cells in young *WRNexo-null* (C) and young wild-type midguts (B).

(D, E) Immunofluorescence images of DAPI-stained whole guts. The length of the *WRNexo-null* midguts (E) was similar to that of the wild-type midguts (D) when flies were young (7 days).

(F) Quantification of the pH3^+^ cells in young (7 days) midguts from control flies (carrying *esg-GFP/+* as control) and *WRNexo-null* flies (*esg-GFP/+; WRNexo^10^/ WRNexo^12^* and *esg-GFP/+; WRNexo^10^/WRNexo^15^*). Each dot represents one midgut, n is as indicated.

(G to I) Immunofluorescence images of midguts with *esg*-GFP staining for young (7 days) control (*esg-GFP/+*, G) and young (7 days) *WRNexo-null* flies (*esg-GFP/+; WRNexo^10^/WRNexo^12^* and *esg-GFP/+; WRNexo^10^/WRNexo^15^*, H and I).

(J) Quantification of *esg*-GFP^+^ cells in a 10,000 μm^2^ area of midguts of flies with indicated genotypes from experiments (G to I). Each dot represents a ROI (region of interest). ROI size was 10,000 μm^2^, n is as indicated.

(K) Quantification of flip-out (F/O) lineage tracing to determine clone sizes for midguts of experiments in (L-N). Each dot represents a clone, n is as indicated.

(L-N) Using the flip-out (F/O) lineage tracing of clones driven by the *esg-gal4* system to detect the clone size of control flies (L, *esg*-gal4 only) and flies treated with different types of *WRNexo-RNAi* (M, N) under a permissive temperature for 14 days. The boxed areas in (L-N) are enlarged on the right.

(O) Relative mRNA fold change of *WRNexo* in *Drosophila* carrying two different *tub-Gal4-driven WRNexo RNAi* lines. The *WRNexo* expression levels in *Drosophila* with different RNAi are plotted relative to levels in control flies (*tub-Gal4/+*), set to 1. Error bars indicate the standard deviation (SD) of three independent experiments.

DAPI-stained nuclei (blue). Scale bars represent 10 μm in B, C, 200 μm in D, E, 25 μm in G-I and enlarged pictures on the right in L-N, and 200 μm in left-hand images of L-N. Error bars represent SD. Student’s t-tests, **p* < 0.05, ***p* < 0.01, ****p* < 0.001, *****p* < 0.0001, and NS (non-significant) represents *p* > 0.05. Underlying data and statistical analysis in S2 Data.
