# Supplementary material for: Werner syndrome exonuclease promotes gut regeneration and causes age-associated gut hyperplasia in Drosophila
Source: PLoS Biol. 2025 Apr 22;23(4):e3003121. doi: 10.1371/journal.pbio.3003121 (PMC12013949; doi:10.1371/journal.pbio.3003121)
Supplement: S3 Fig — (DOCX) [file pbio.3003121.s003.docx]

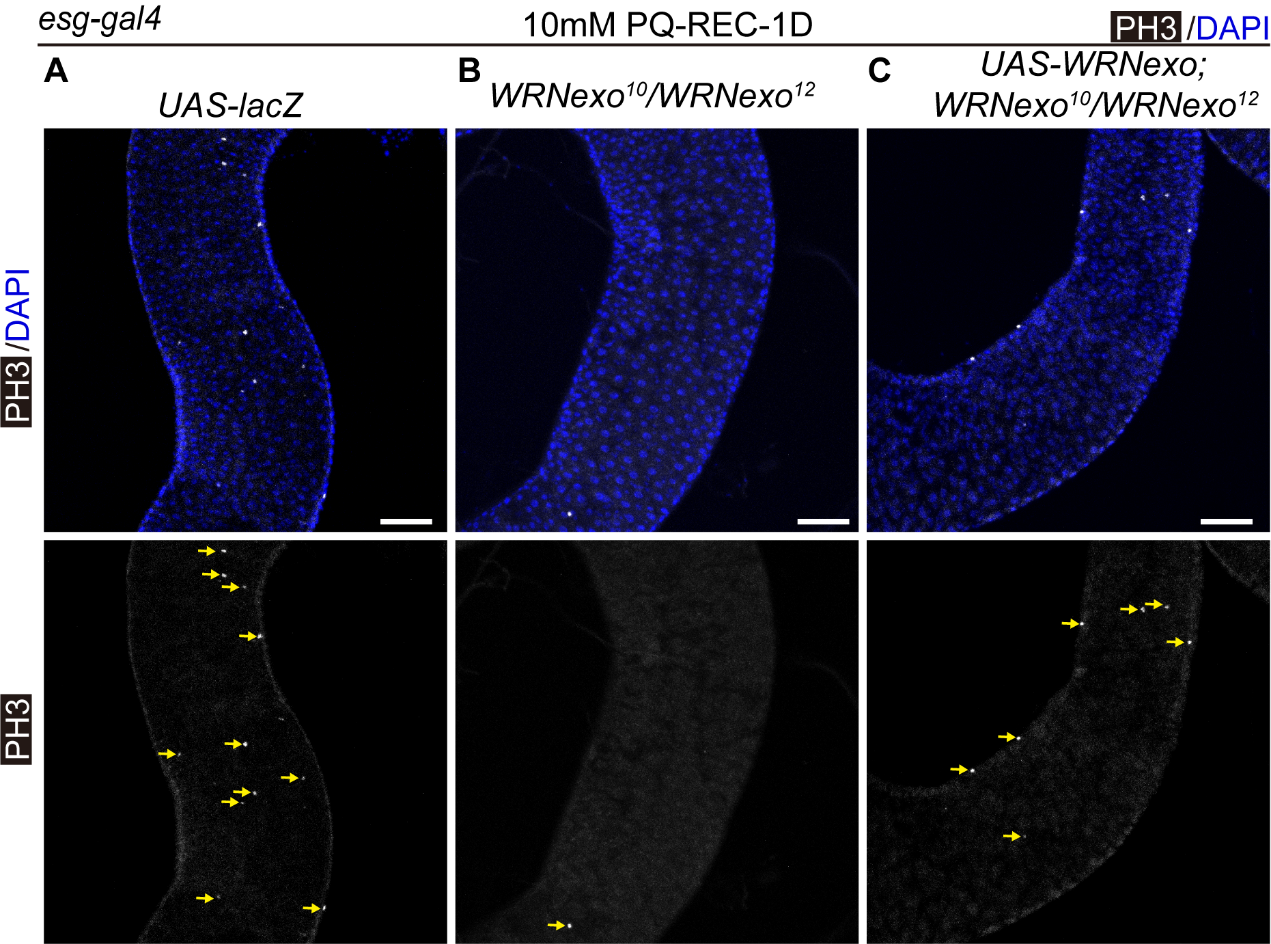


**S3 Fig.** **WRNexo functions cell-autonomously in ISCs to promote ISC proliferation, related to Fig 3.**

(A, B, C) Representative images of DAPI and pH3^+^ staining in midguts from control

(A), *WRNexo-null* (B), and combined *WRNexo*-overexpressing with *WRNexo-null* (C) flies. Flies were treated with PQ-REC-1D. Single-channel images of midguts with pH3^+^ immunostaining are shown in the lower panels.

DAPI-stained nuclei (blue). Scale bars represent 75 μm in A-C.
