# Supplementary material for: Werner syndrome exonuclease promotes gut regeneration and causes age-associated gut hyperplasia in Drosophila
Source: PLoS Biol. 2025 Apr 22;23(4):e3003121. doi: 10.1371/journal.pbio.3003121 (PMC12013949; doi:10.1371/journal.pbio.3003121)
Supplement: S4 Fig — Underlying data and statistical analysis in S4 Data. (DOCX) [file pbio.3003121.s004.docx]

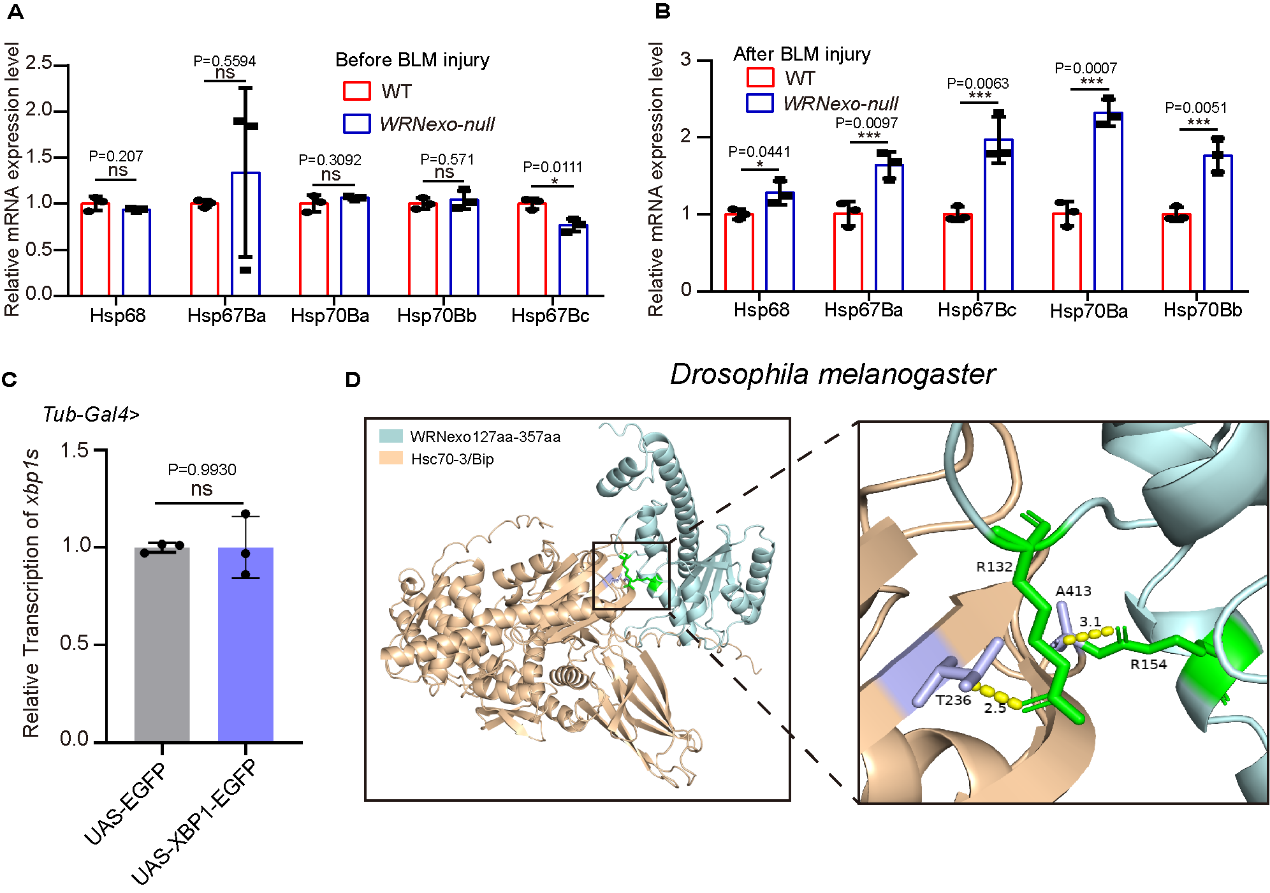
 **S4 Fig. WRNexo protein directly interacts with protein Hsc70-3/Bip, related to Fig 4 and Fig 5.**

(A-B) Relative mRNA fold changes of *Hsp68*, *Hsp67Ba*, *Hsp67Bc*, *Hsp70Ba*, and *Hsp70Bb* in *WRNexo-null* midguts compared to WT midguts before (A) and after (B) BLM-REC-1D treatment. The increases in expression are plotted relative to levels in controls (WT flies’ midguts), set to 1. Error bars indicate the standard deviation (SD) of three independent experiments.

(C) Relative mRNA fold changes of *Xbp1s* in *UAS-XBP1-EGFP* midguts compared to *UAS-EGFP* midguts. Expression levels are normalized to controls (UAS-EGFP), which are set to 1.

(D) Structure of *Drosophila* WRNexo (cyan) and Hsc70-3/Bip (yellow) has been predicted in Alphafold. *Drosophila* WRNexo (127aa-357aa) and *Drosophila* Hsc70-3/Bip have integrally secondary structures. The motifs of *Drosophila* WRNexo and Hsc70-3/Bip were predicted in <https://neurosnap.ai/service/AlphaFold2>.

Error bars represent SD. Student’s t-tests, **p* < 0.05, ****p* < 0.001, and NS (non-significant) represents *p* > 0.05. Underlying data and statistical analysis in S4 Data.
