# Supplementary material for: Werner syndrome exonuclease promotes gut regeneration and causes age-associated gut hyperplasia in Drosophila
Source: PLoS Biol. 2025 Apr 22;23(4):e3003121. doi: 10.1371/journal.pbio.3003121 (PMC12013949; doi:10.1371/journal.pbio.3003121)
Supplement: S5 Fig — Underlying data and statistical analysis in S6 Data. (DOCX) [file pbio.3003121.s005.docx]

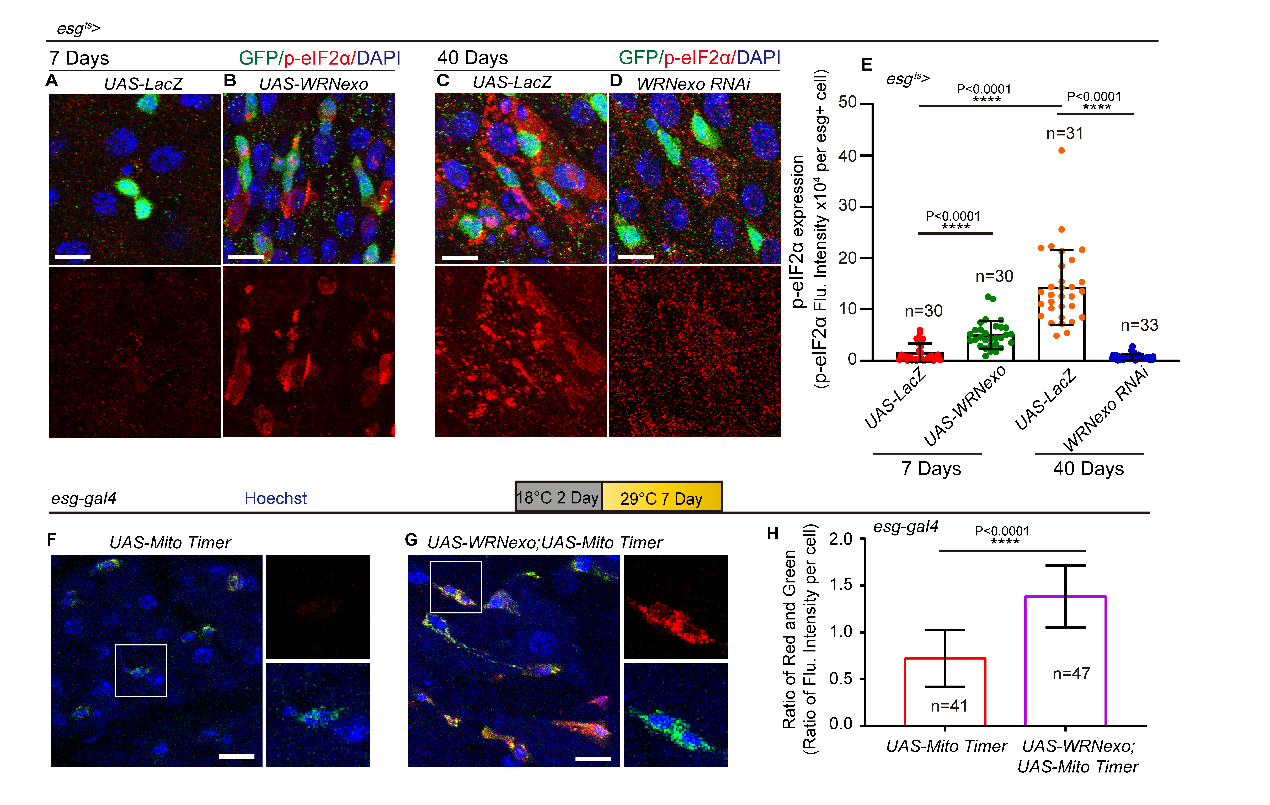


**S5 Fig. WRNexo regulates the cellular redox state and modulates ISC proliferation by UPR^ER^ pathway, related to Fig 6.**

(A, B) Representative images of the young midguts of flies carrying *esg^ts^-GAL4-driven UAS-LacZ* (control, A) and *UAS-WRNexo* (B) under permissive temperature, stained with p-eIF2α and GFP. The lower panel shows single channel of p-eIF2α.

(C, D) Representative images of the old midguts of flies carrying *esg^ts^-GAL4-driven UAS-LacZ* (control, F) and *WRNexo RNAi* (G) under permissive temperature, stained with p-eIF2α and GFP. The lower panel shows single channel of p-eIF2α.

(E) Quantification of p-eIF2α fluorescence intensity per *esg*^+^ cell from experiments (A-D); each dot represents one *esg*^+^ cell.

(F, G) Immunofluorescence images of midguts of flies carrying *esg-GAL4-driven UAS-MitoTimer* (F, control) and *esg-GAL4-driven UAS-MitoTimer* combined with *WRNexo* cDNA expression (G). Hoechst-stained nuclei (blue).

(H) Quantification of the ratio of red and green fluorescence intensity per cell from experiments (F, G). n is as indicated.

Hoechst- and DAPI-stained nuclei (blue). Scale bars represent 5 μm in A-D and F-G. Error bars represent SD. Student’s t-tests, **p* < 0.05, ***p* < 0.01, ****p* < 0.001, *****p* < 0.0001, and NS (non-significant) represents *p* > 0.05. Underlying data and statistical analysis in S6 Data.
