# Supplementary material for: Werner syndrome exonuclease promotes gut regeneration and causes age-associated gut hyperplasia in Drosophila
Source: PLoS Biol. 2025 Apr 22;23(4):e3003121. doi: 10.1371/journal.pbio.3003121 (PMC12013949; doi:10.1371/journal.pbio.3003121)
Supplement: S6 Fig — Underlying data and statistical analysis in S6 Data. (DOCX) [file pbio.3003121.s006.docx]

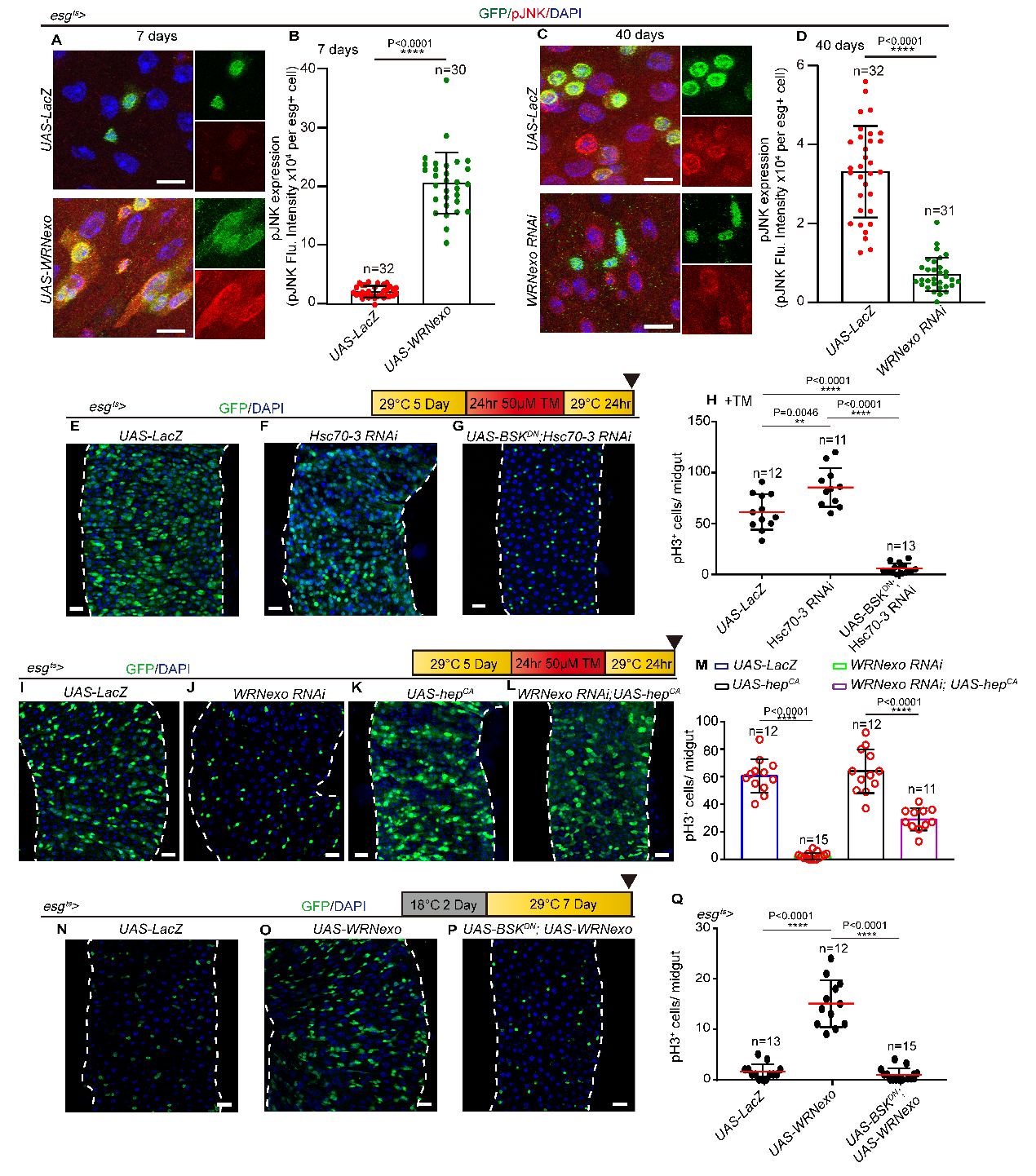


**S6 Fig. WRNexo regulates ISC proliferation by Hsc70-3 and JNK signaling,** **related to Fig 6.**

(A) Representative images of the expression of pJNK in midguts from young flies carrying *esg^ts^-GAL4-driven UAS-LacZ* (control, upper panel) and *UAS-WRNexo* (lower panel) under a permissive temperature.

(B) Quantification of pJNK fluorescence intensity per *esg*^+^ cell from experiments (A); each dot represents one *esg*^+^ cell.

(C) Representative images of the expression of pJNK in midguts from old flies carrying *esg^ts^-GAL4-driven UAS-LacZ* (control, upper panel) and *WRNexo RNAi* (lower panel) under a permissive temperature.

(D) Quantification of pJNK fluorescence intensity per *esg*^+^ cell from experiments (C); each dot represents one *esg*^+^ cell.

(E-G) Immunofluorescence images of the midgut of flies carrying *esg^ts^-GAL4-driven UAS-LacZ* (control, E), *Hsc70-3 RNAi* (F), or *Hsc70-3 RNAi* with *UAS-Bsk^DN^* (G) under TM-REC-1D treatment.

(H) Quantification of pH3^+^ cells from whole midguts of experiments in (E-G). Each dot represents a midgut, n is as indicated.

(I-L) Immunofluorescence images of the midguts of flies carrying *esg^ts^-GAL4-driven UAS-LacZ* (control, I), *WRNexo RNAi* (J), *UAS-hep^CA^* (K), or *WRNexo RNAi* with *UAS-hep^CA^* (L) under TM-REC-1D treatment.

(M) Quantification of pH3^+^ cells from whole midguts of experiments in (I-L). Each red circle represents a midgut, n is as indicated.

(N-P) Representative images of the midgut of flies carrying *esg^ts^-GAL4-driven UAS-LacZ* (control, N), *UAS-WRNexo* (O), or *UAS-WRNexo* with *UAS-Bsk^DN^* (P) under a permissive temperature.

(Q) Quantification of pH3^+^ cells from whole midguts of experiments in (N-P). Each dot represents a midgut, n is as indicated.

DAPI-stained nuclei (blue). Scale bars represent 5 μm in A and C, 25 μm in E-G, I-L and N-P. Error bars represent SD. Student’s t-tests, **p* < 0.05, ***p* < 0.01, ****p* < 0.001, *****p* < 0.0001, and NS (non-significant) represents *p* > 0.05. Underlying data and statistical analysis in S6 Data.
