# Supplementary material for: Werner syndrome exonuclease promotes gut regeneration and causes age-associated gut hyperplasia in Drosophila
Source: PLoS Biol. 2025 Apr 22;23(4):e3003121. doi: 10.1371/journal.pbio.3003121 (PMC12013949; doi:10.1371/journal.pbio.3003121)
Supplement: S7 Fig — Underlying data and statistical analysis in S7 Data. (DOCX) [file pbio.3003121.s007.docx]

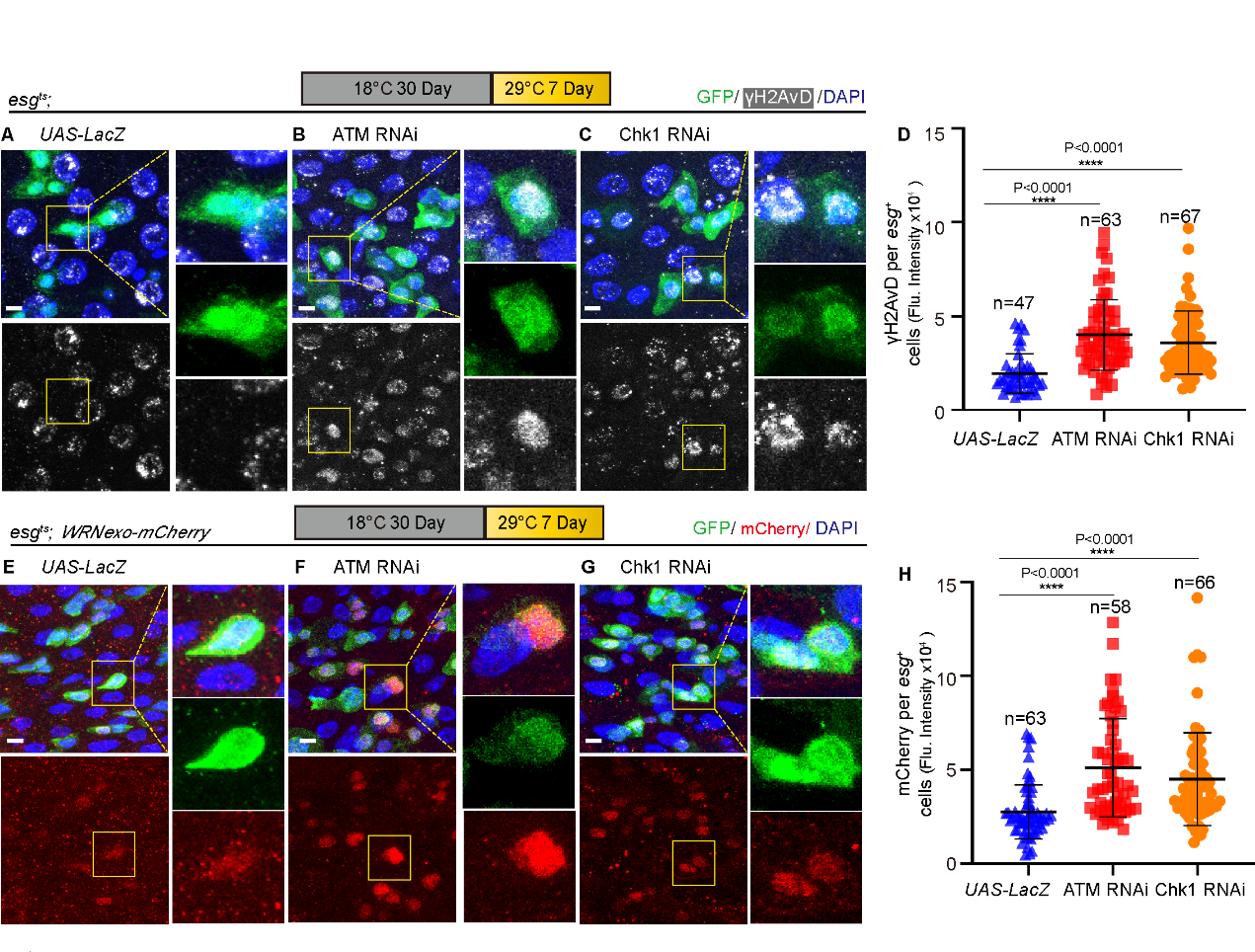


**S7 Fig. DNA double strands break induces WRNexo expression, related to Fig 7.**

(A-C) Immunofluorescence images of the midguts stained for γH2AvD and GFP of flies carrying *esg^ts^-GAL4*-driven *UAS-LacZ* (control, A), *ATM RNAi* (B), and *Chk1 RNAi* (C). Flies were raised on regular food and at a repressive temperature (18℃) for 30 days after eclosion, then shifted to a permissive temperature (29℃) for 7 days with normal conditions before dissection.

(D) Quantification of the fluorescence intensity of γH2AvD midgut-derived from experiments in (A-C). Each dot represents an *esg*-GFP^+^ cell, n is as indicated.

(E-G) Immunofluorescence images of the midguts stained for γH2AvD and GFP of flies carrying *esg^ts^-GAL4*-driven *UAS-LacZ* combined with *WRNexo-mCherry* (control, E), *ATM RNAi* (F), and *Chk1 RNAi* (G). Flies were raised on regular food and at a repressive temperature (18℃) for 30 days after eclosion, then shifted to a permissive temperature (29℃) for 7 days with normal conditions before dissection.

(H) Quantification of the fluorescence intensity of mCherry midgut-derived from experiments in (E-G). Each dot represents an *esg*-GFP^+^ cell, n is as indicated.

DAPI-stained nuclei (blue). Scale bars represent 5 μm in A-C and E-G. Error bars represent SD. Student’s t-tests, **p* < 0.05, ***p* < 0.01, ****p* < 0.001, *****p* < 0.0001, and NS (non-significant) represents *p* > 0.05. Underlying data and statistical analysis in S7 Data.
