# Supplementary material for: Werner syndrome exonuclease promotes gut regeneration and causes age-associated gut hyperplasia in Drosophila
Source: PLoS Biol. 2025 Apr 22;23(4):e3003121. doi: 10.1371/journal.pbio.3003121 (PMC12013949; doi:10.1371/journal.pbio.3003121)

Source data for Fig 1D

WRN expression in Human Colon

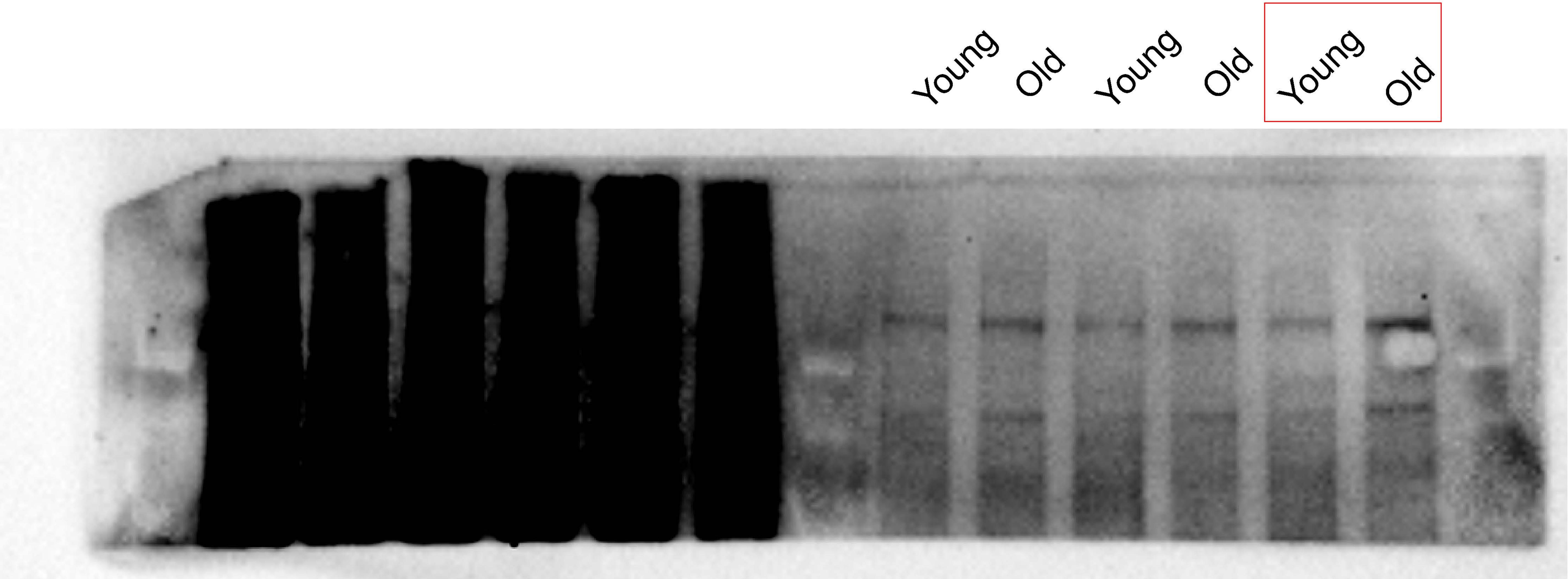

Tublin

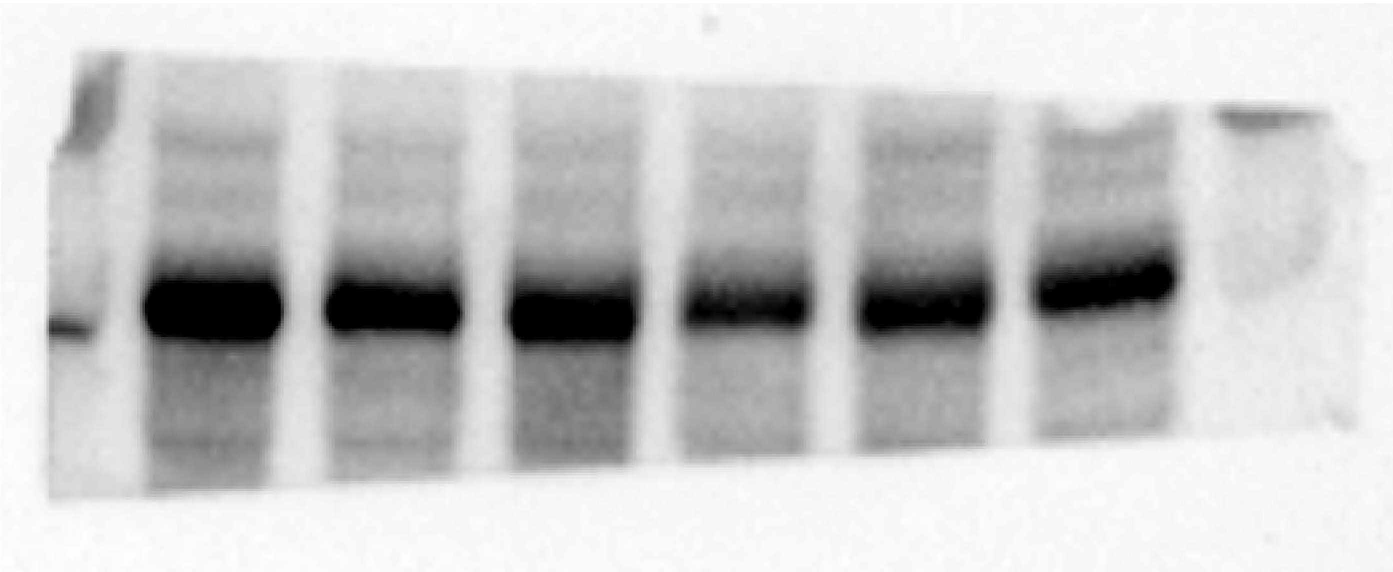

Source data for Fig 1K

WRN expression in Mouse crypts

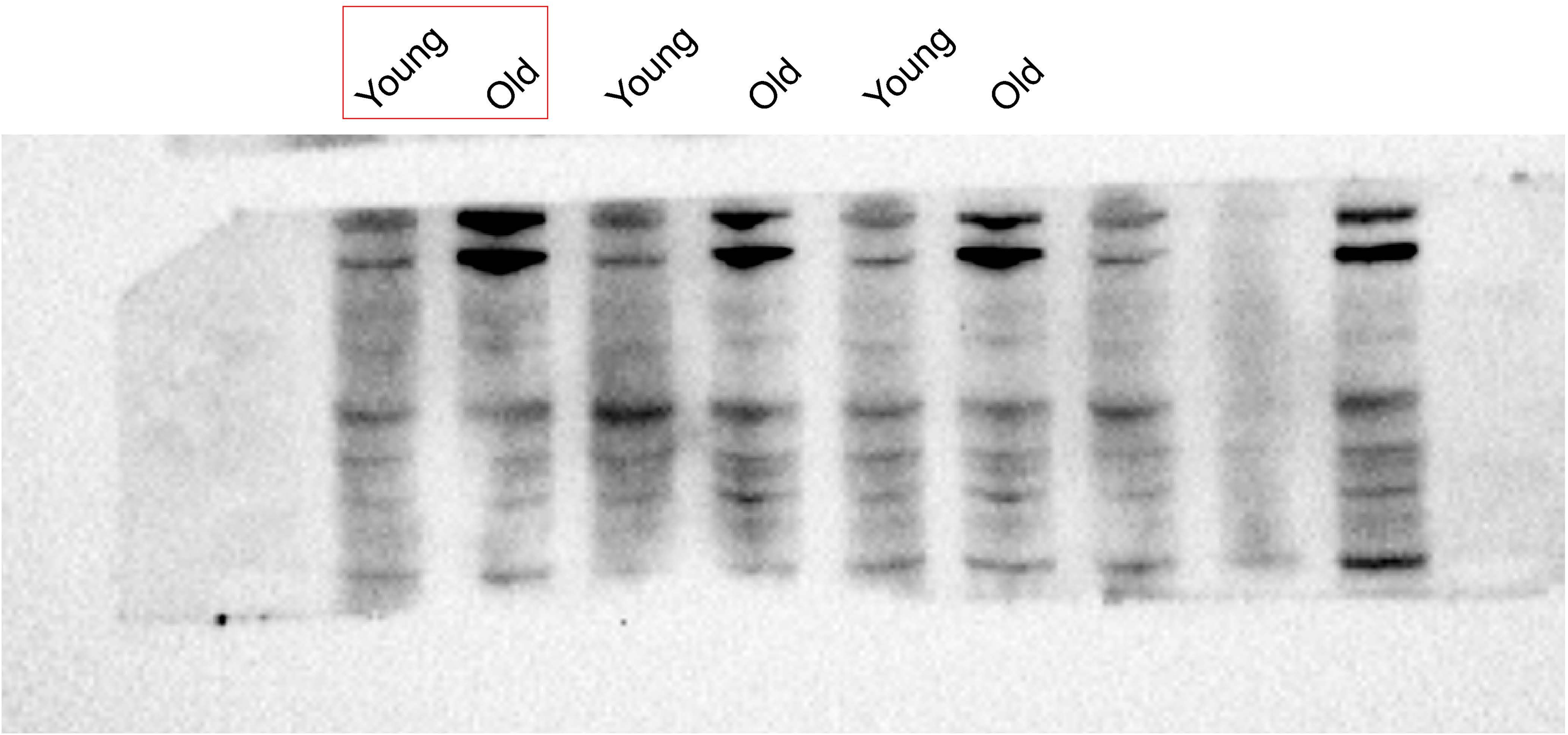

Tublin

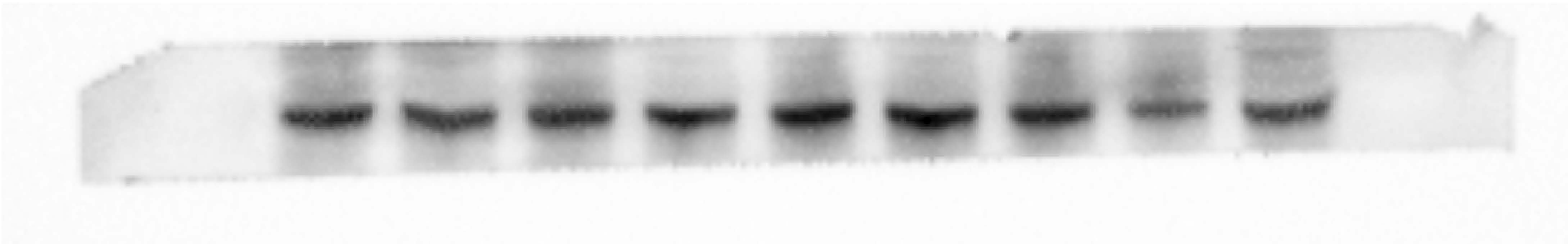

Source data for Fig 5D

1 2

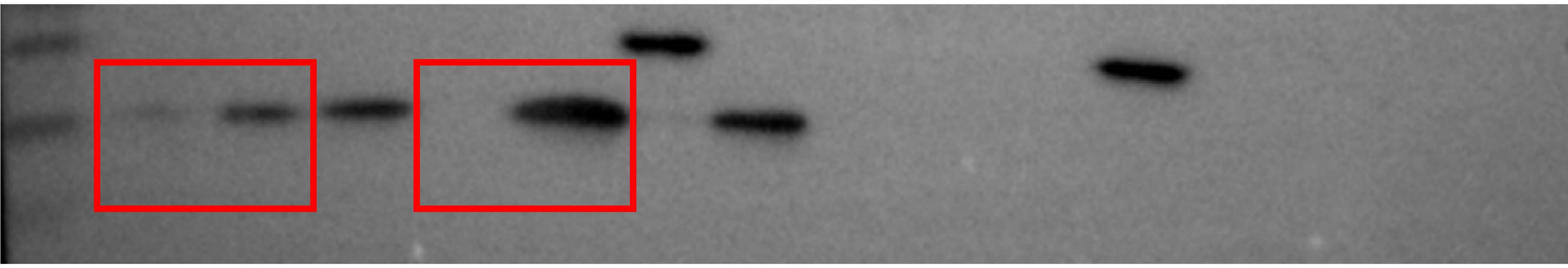

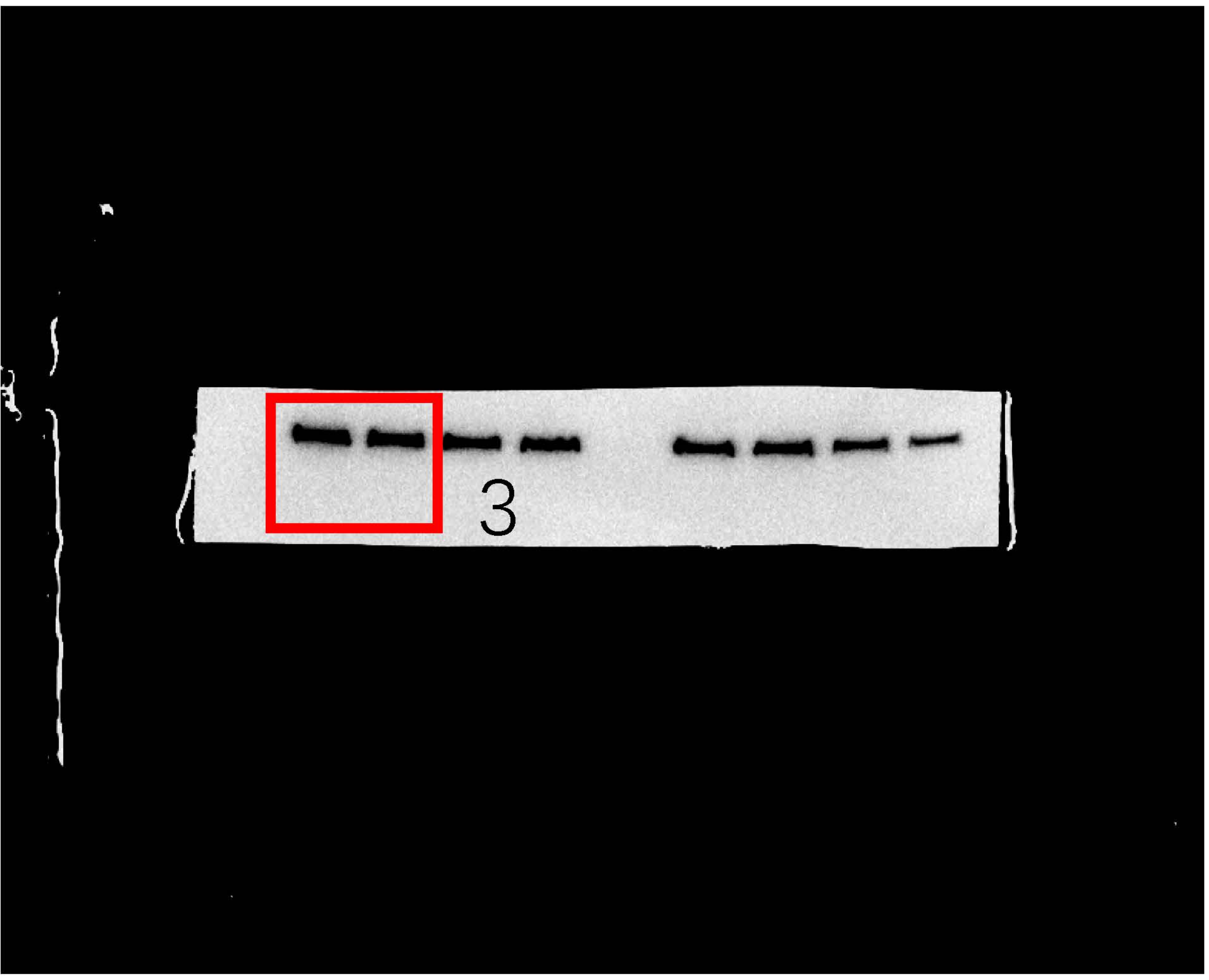

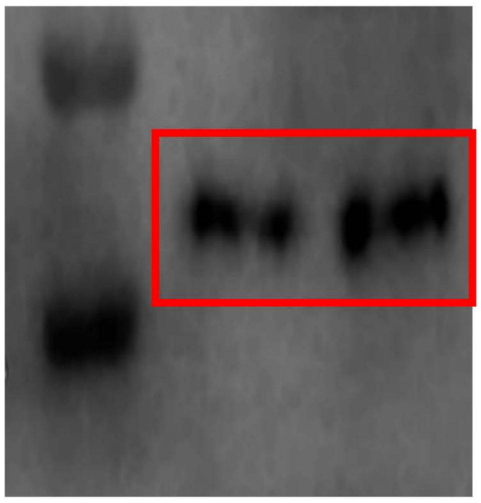

4

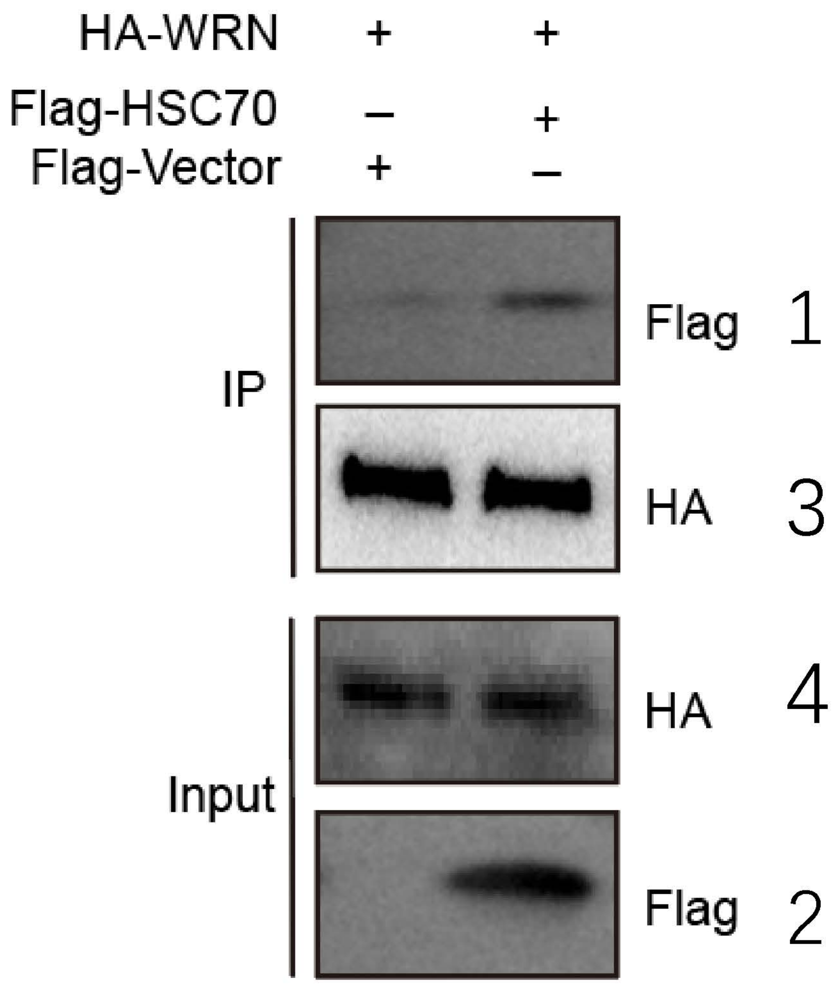

Source data for Fig 5E

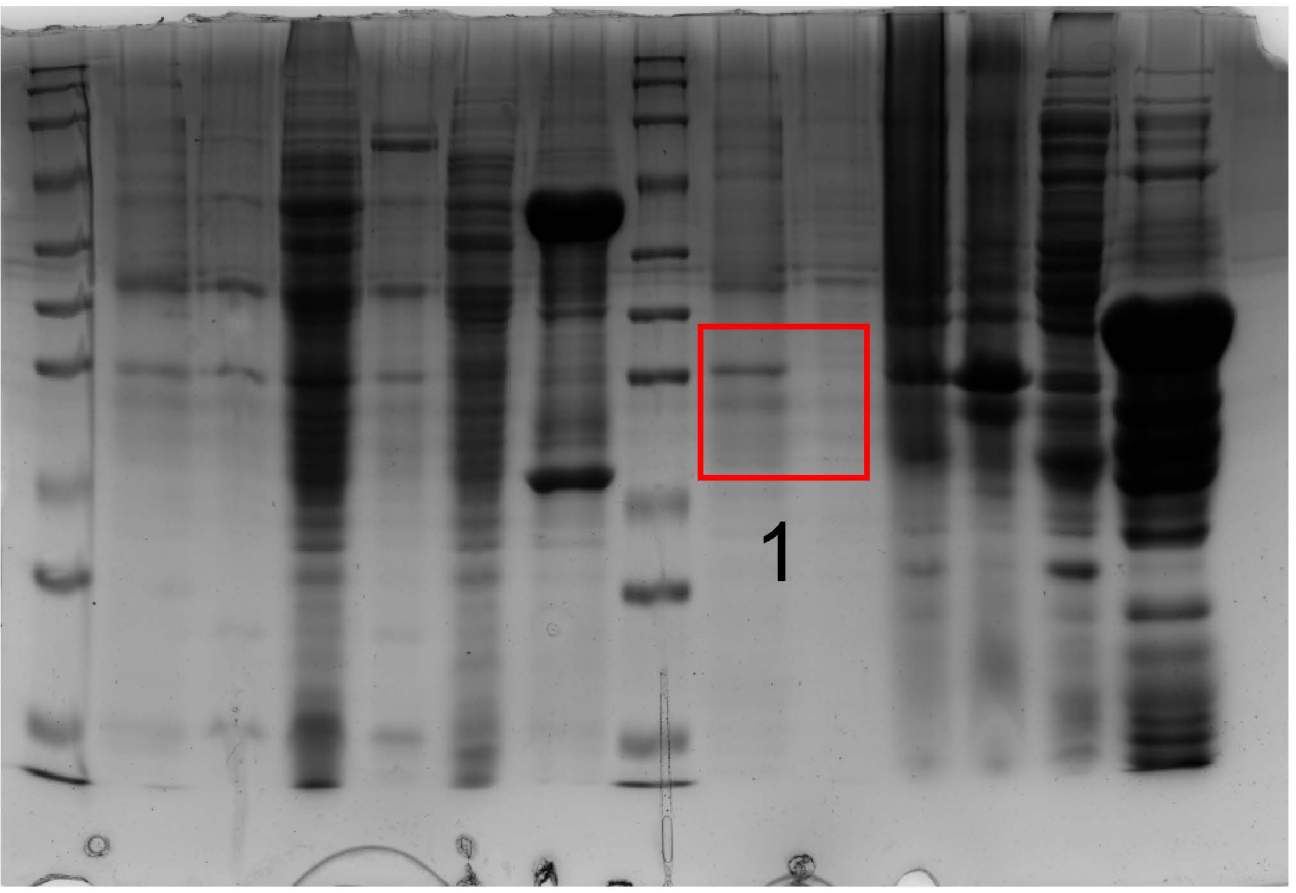

2

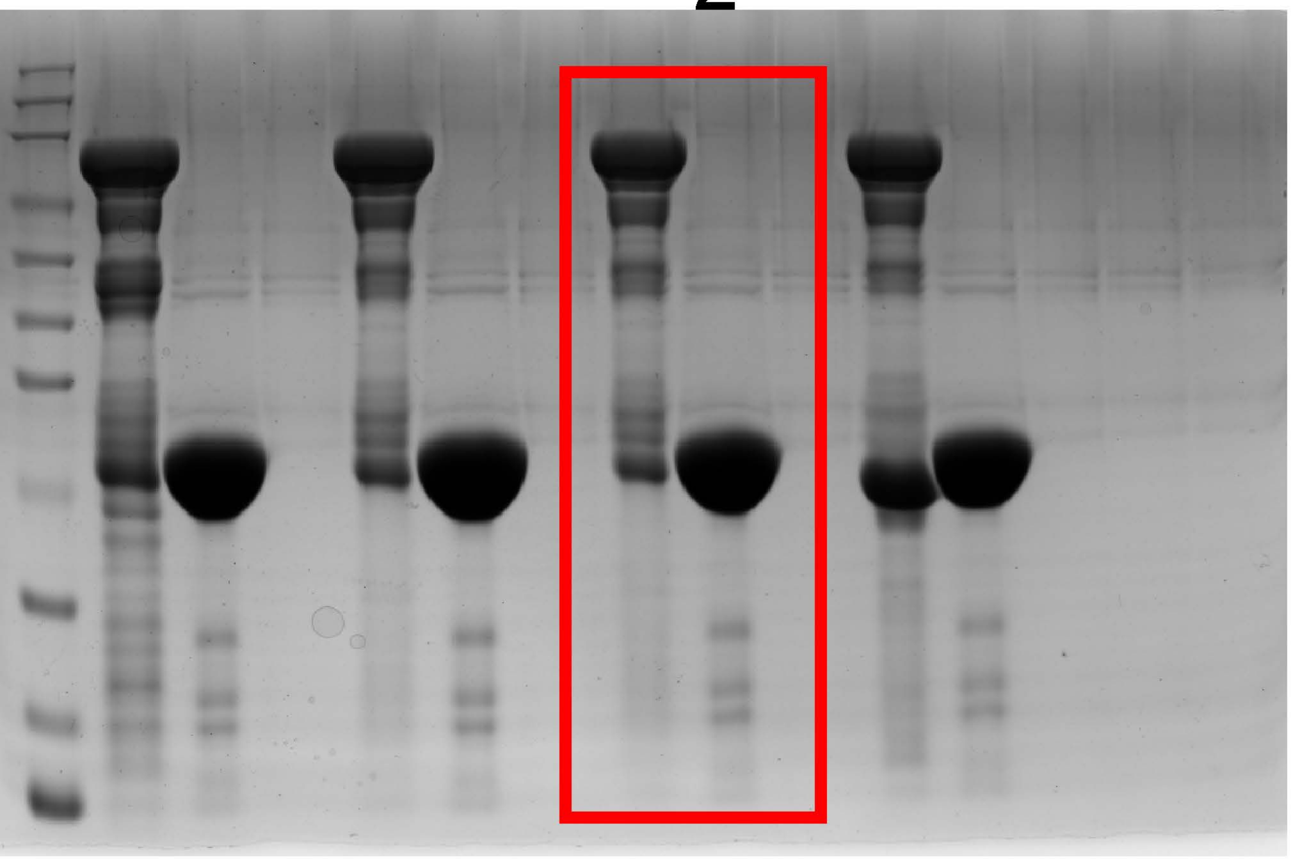

Coomassie blue

1

3

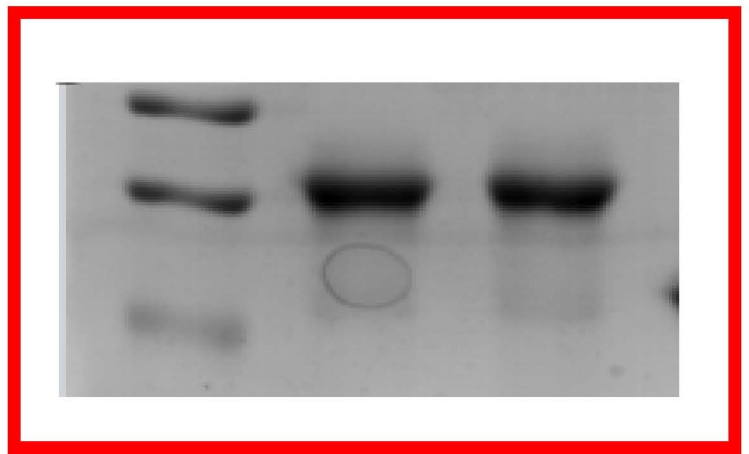

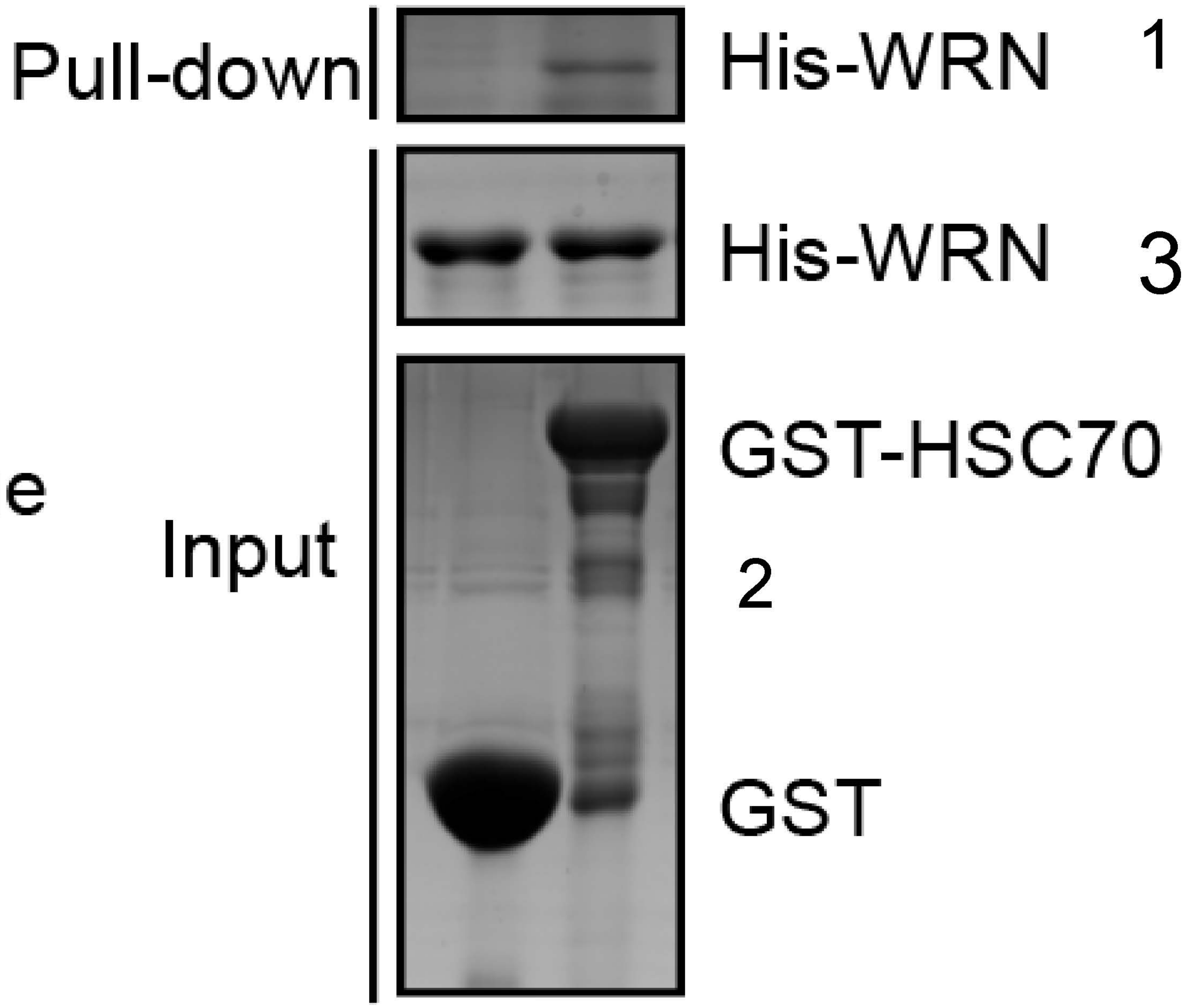

Source data for Fig 6J

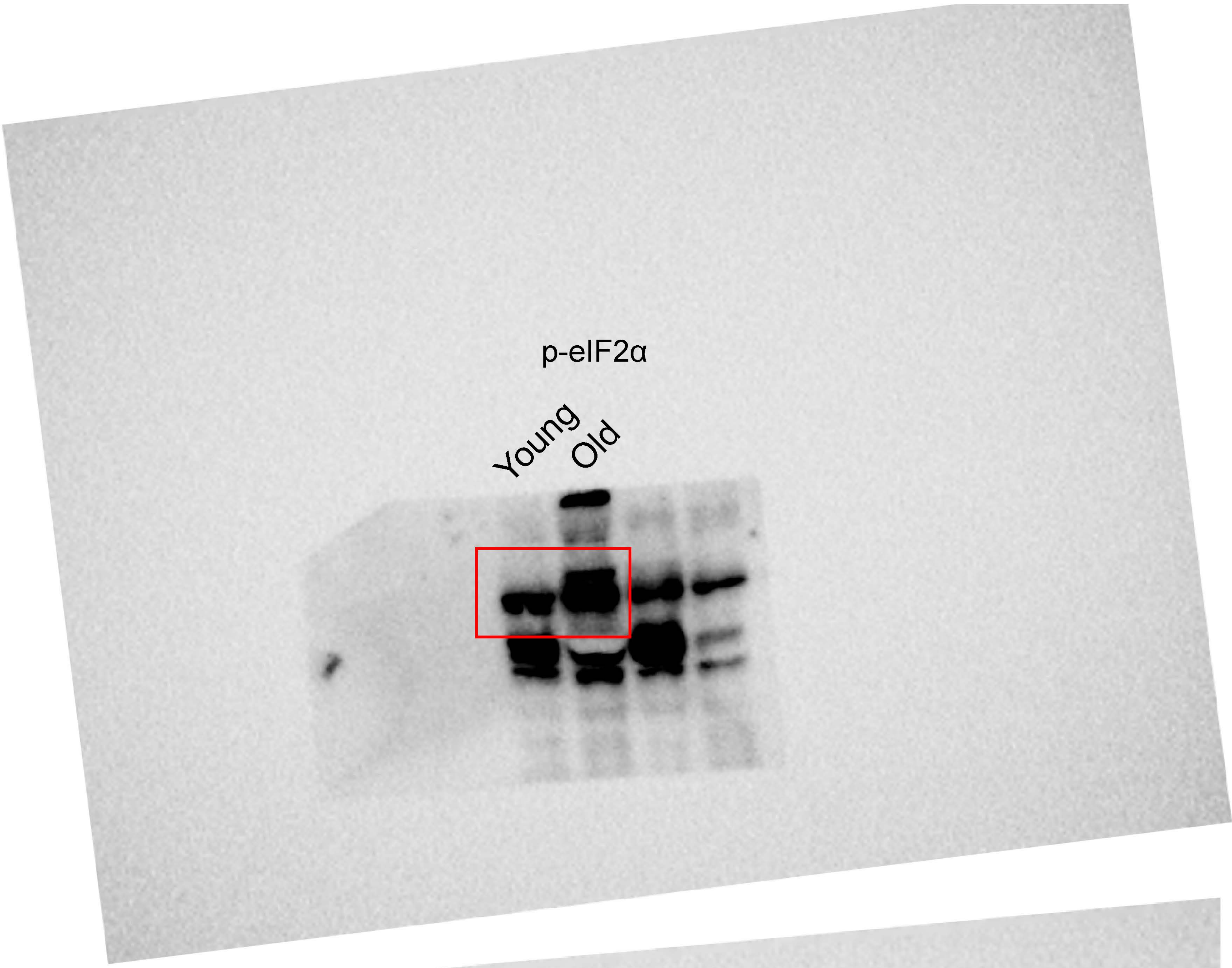

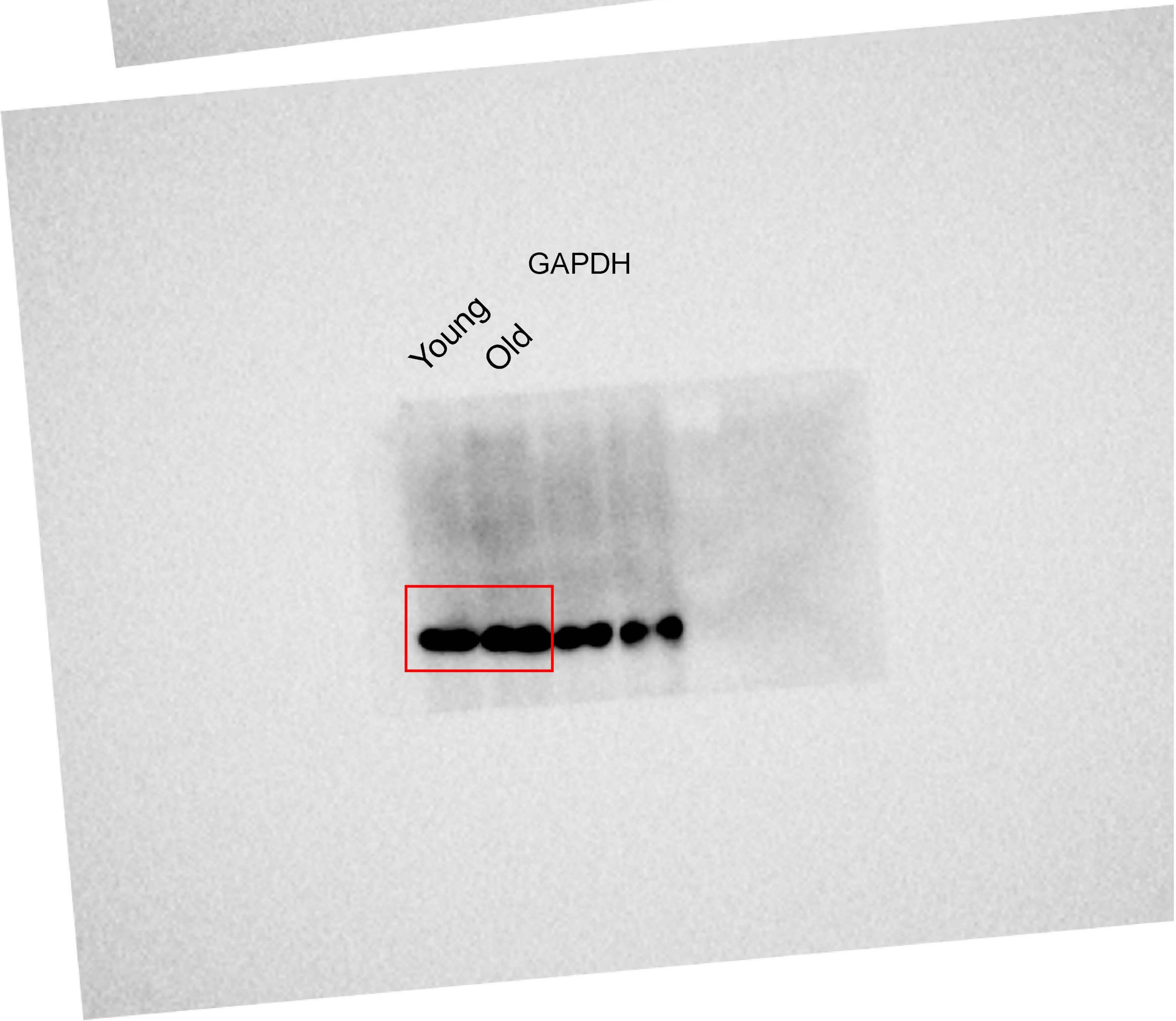

Supplement: S1 Raw Images — (PDF) [file pbio.3003121.s021.pdf]
