# Supplementary material for: Werner syndrome exonuclease promotes gut regeneration and causes age-associated gut hyperplasia in Drosophila
Source: PLoS Biol. 2025 Apr 22;23(4):e3003121. doi: 10.1371/journal.pbio.3003121 (PMC12013949; doi:10.1371/journal.pbio.3003121)
Supplement: Attachment — Submitted filename: PLOS BIOL WRN ext review.docx [file pbio.3003121.s023.docx]

It is a worthwhile study to investigate the role of WRN in the aging process in the gut, but this is not as novel as they claim and there has been many studies of WRN expression with aging in humans and model systems.

There are many semantic errors in the text and it has not been proofed for English language.

The work represents an overload of diverse data that represent different aspects and do not all fit together. The quality of the experiments are generally high but too many diverse things are being explored and often not sufficiently in depth.

The observations of an association between Hsc70 and WRN exo (Fig 5) are potentially interesting, and they need to follow that up to determine if there is a functional interaction between these two proteins. If Hsc70 fragments stimulate the exonuclease activity in vitro, it would strongly support their finding.

The DNA damage experiments are somewhat superficial and do not explain causal association. Also they, use UV and claim it induces DNA double strand breaks. That is not the case.

The use of human colon samples is not so relevant here. As they point out, the Drosophila WRN only has Exo activity and is therefore quite different from the human, which also has helicase activity and there are many studies with human WRN implicating it in replication, but mainly via helicase function.
