# Supplementary material for: Werner syndrome exonuclease promotes gut regeneration and causes age-associated gut hyperplasia in Drosophila
Source: PLoS Biol. 2025 Apr 22;23(4):e3003121. doi: 10.1371/journal.pbio.3003121 (PMC12013949; doi:10.1371/journal.pbio.3003121)
Supplement: Attachment — Submitted filename: Response to Reviewers.docx [file pbio.3003121.s025.docx]

Reviewers' comments

Rev. 1:

It is a worthwhile study to investigate the role of WRN in the aging process in the gut, but this is not as novel as they claim and there has been many studies of WRN expression with aging in humans and model systems.

*Response:*

*Thank you for the reviewer’s comments. Over the past decade, research on WRN has primarily focused on its role in Werner syndrome and in cultured cells(Fang et al., 2019; Lautrup et al., 2019; Shamanna et al., 2017; Zhang et al., 2015), with limited understanding of its function in natural aging in vivo. In our study, using the Drosophila gut as a model, we unexpectedly found that WRN expression increases during natural aging, a finding that contradicts prior expectations derived from studies of WRN deficiency, which is known to result in premature aging(Kudlow et al., 2007). Furthermore, WRNexo is predominantly expressed in intestinal stem cells (ISCs) and enteroblasts (EBs), highlighting the need for further exploration of WRNexo's role in stem cell regulation during aging.* *The WRN protein is known to have two functional domains: an exonuclease and a RecQ helicase. While its helicase activity has been extensively studied and linked to genomic stability, evidence suggests it cannot fully explain all observed phenotypes. For instance, helicase inhibitors do not provoke hypersensitivity in WRN+/+ cells, and mutations solely affecting helicase activity fail to recapitulate Werner syndrome(Kamath-Loeb et al., 2017; Poot et al., 2001). These findings point to the important, yet understudied, role of WRN exonuclease activity, warranting further investigation. Interestingly, Drosophila WRN exhibits only exonuclease activity, making it an ideal model to investigate the specific role of this enzymatic function in aging.*

*These findings provide new insights into WRN's role in the natural aging process and its potential relevance to intestinal stem cell function. To address the reviewer’s concern, we have revised the introduction and discussion to better summarize the current state of WRN research and to avoid overstating our findings in the revised manuscript.*

There are many semantic errors in the text and it has not been proofed for English language.

*Response:*

*We apologize for the semantic errors in the manuscript. We have carefully reviewed and corrected the expression errors in the text. To further enhance the language quality, the revised manuscript was proofread by a native English-speaking editor from MogoEdit.com. We are confident that these revisions have ensured the manuscript is accurate and well-expressed in English.*

*A certification from MogoEdit.com*

The work represents an overload of diverse data that represent different aspects and do not all fit together. The quality of the experiments are generally high but too many diverse things are being explored and often not sufficiently in depth.

*Response:*

*Thank you for your valuable feedback. We appreciate the opportunity to clarify our research rationale and narrative structure. Our manuscript aims to explore the distinct roles of WRNexo in maintaining gut homeostasis, particularly in response to injury, and its contribution to aging, utilizing models such as, BLM-induced damage, PQ-induced oxidative stress, and natural aging. We have also included preliminary validations in mouse and human samples to explore the broader applicability of our findings. We acknowledge the concern regarding the overload of diverse data and the perceived lack of depth in some sections.* *In response, we have carefully revised the manuscript to enhance focus and coherence. To address the concern about depth, we have added lifespan assays to provide stronger evidence for WRNexo’s role in aging regulation (Fig. 6, E and F). Additionally, we conducted experiments modulating WRNexo and Hsc70-3/Bip expression to demonstrate their functional interaction in regulating ER stress by assessing Xbp1 splicing (ER stress reporter: Xbp1-EGFP), further supporting the mechanistic significance of their binding (Fig.5, F to K). To ensure that the central theme—the role of WRNexo in gut regeneration and aging—is clearly emphasized, we have removed data and discussions that are less relevant. Furthermore, we have reorganized the presentation of results to provide a more logical and cohesive narrative, making the findings easier to follow. The language throughout the manuscript has also been refined to eliminate redundancy and improve clarity, ensuring that the results are presented concisely and precisely.*

*We believe these adjustments address the reviewer’s concerns and greatly appreciate your constructive feedback, which has guided us in enhancing our work.*

*(Fig. 6, E and F)*

*(Fig.5, F to K)*

The observations of an association between Hsc70 and WRN exo (Fig 5) are potentially interesting, and they need to follow that up to determine if there is a functional interaction between these two proteins. If Hsc70 fragments stimulate the exonuclease activity in vitro, it would strongly support their finding.

*Response:*

*Thank you for the reviewer’s valuable suggestion. We agree that demonstrating the functional interaction between WRNexo and Hsc70-3/Bip is crucial. Using mass spectrometry (IP-MS), AlphaFold prediction, immunoprecipitation (co-IP), and pull-down assays, we identified and validated Hsc70-3/Bip as a binding partner of WRNexo (Fig. 5, A to E, and Supplemental Fig. S4C). Mechanistically, WRNexo regulates ER stress and ISC proliferation by binding to and inhibiting Hsc70-3/Bip.* *To further elucidate this interaction, genetic experiments revealed that Hsc70-3/Bip knockdown phenocopied WRNexo overexpression, promoting UPR^ER^ signaling and inducing Xbp1 splicing, as evidenced by the nuclear localization of XBP1-EGFP (Fig. 5, F to H). Conversely, co-expression of Hsc70-3/Bip and WRNexo attenuated this phenotype, suggesting that WRNexo modulates UPR^ER^ signaling through its interaction with Hsc70-3/Bip (Fig. 5, F to K). These findings underscore the critical role of Hsc70-3/Bip in mediating WRNexo’s effects.*

*While Drosophila WRN exhibits only exonuclease activity(Saunders et al., 2008), our study focuses on in vivo functional mechanisms rather than biochemical details.* *Investigating whether Hsc70 fragments stimulate WRNexo’s exonuclease activity in vitro is an intriguing avenue for future research. We have revised the manuscript to include these findings and address the limitations of our current work, highlighting the importance of further studies to explore this question.*

*(Fig.5, A to E)*

*(Supplemental Fig. S4C)*

*(Fig.5, F to K)*

The DNA damage experiments are somewhat superficial and do not explain causal association. Also they, use UV and claim it induces DNA double strand breaks. That is not the case.

*Response:*

*Thank you for your insightful comments regarding the DNA damage experiments. We apologize for the imprecise description in the original manuscript. You are correct that UV irradiation primarily induces pyrimidine dimers and single-strand breaks (SSBs), while double-strand breaks (DSBs) are secondary events resulting from oxidative stress or repair processes. To address this, we have removed the UV experiments and replaced them with X-ray irradiation, which directly induces DSBs (Fig.7, K to N).* *Additionally, we performed experiments manipulating DNA damage repair genes, such as ATM and Chk1, to further validate the link between DNA damage and WRNexo expression. Specifically, we observed that knocking down ATM or Chk1 in middle-aged flies, which impairs DNA repair capacity, significantly increased WRNexo expression in ISCs, consistent with our X-ray results (Supplemental Fig. S7 A to H).*

*While we provide evidence that DNA damage is a significant contributing factor to WRNexo upregulation during aging, we recognize that other mechanisms may also play a role. Aging is a highly complex process influenced by multiple factors, such as increased ROS production and ER stress, which may also regulate WRNexo expression. We have revised the discussion sections to clarify these points and to emphasize the need for further studies to investigate the interplay between these factors.*

*(Fig.7, K to N)*

*(Supplemental Fig. S7 A to H)*

The use of human colon samples is not so relevant here. As they point out, the Drosophila WRN only has Exo activity and is therefore quite different from the human, which also has helicase activity and there are many studies with human WRN implicating it in replication, but mainly via helicase function.

***Response:***

*Thank you for the reviewer’s insightful comments. We fully understand the concerns raised. The inclusion of human colon samples in our study aimed to demonstrate the significant upregulation of WRN expression in aging and aging-related diseases, such as colorectal cancer. This finding underscores the potential importance of WRN in human aging. Additionally, we observed increased ER stress in aging human samples, which aligns with our findings in Drosophila and supports the relevance of WRN-mediated mechanisms in regulating ISC aging across species.*

*We acknowledge that human WRN possesses both helicase and exonuclease (Exo) activities,* *whereas Drosophila WRN is limited to Exo activity. While helicase activity has been extensively studied and linked to genomic stability, evidence suggests it cannot fully explain all observed phenotypes. For instance, helicase inhibitors do not provoke hypersensitivity in WRN+/+ cells, and mutations solely affecting helicase activity fail to recapitulate Werner syndrome(Kamath-Loeb et al., 2017; Poot et al., 2001). These findings point to the important, yet understudied, role of exonuclease activity, which is the focus of our study. This difference is an important limitation, as WRN’s helicase activity may contribute to its role in aging in humans. We have addressed this limitation in the revised discussion and limitations sections of the manuscript.*

*The use of human and mouse samples allowed us to explore the potential evolutionary conservation of WRN’s role in aging and ER stress regulation. However, we understand the reviewer’s concerns regarding the relevance of human data to this study. If deemed inappropriate, we are willing to remove the results from human samples to maintain the focus and rigor of the research.*

Rev. 2:

In this manuscript, Wu and coworkers examine the role of Werner syndrome exonuclease in natural ageing. This is an interesting topic as the loss-of-function in this exonuclease results in progeria but the role of the protein in normal ageing has not been examined. The authors present data, obtained mostly in Drosophila, to support their model where WRNexo accumulates due to DNA damage in intestinal stem cells (ISCs), modulates the ER unfolded protein response and redox balance in the ISCs and drives intestinal hyperplasia during ageing. In a way, this contrasts to the progeria observed upon loss-of-function of WRN. Overall, the work is interesting and well executed but there are a few aspects of their model that do not appear to fit; I find they do not make sense, at least not to me, and I think they need to be addressed before I can recommend the manuscript for publication in PLoS Biology. These are predominantly to do with the analysis of Xbp1's role:

*Response:*

*We appreciate that the reviewer gives* *positive comments of our study.* *In order to address the issues mentioned by the reviewer, we have provided a detailed explanation of the reviewer’s concerns and outlined our experimental results* *in the current revised version.*

1. Regarding Xbp1 activation: This is usually assessed by looking at splicing of the 1ry transcript, but the authors look at Xbp1 nuclear localization and in the conditions where Xbp1-EGFP is overexpressed. What is this Xbp1-EGFP construct? Is it reporting splicing? In this case this should be made explicit and consistent throughout the text. Additionally, it would be important to look at the splicing of the native transcript in at least one of their experiments, to avoid any potential artefacts of Xbp1-EGFP overexpression.

***Response:***

*Thank you for the reviewer’s comments. We appreciate the opportunity to clarify the Xbp1-EGFP system used in our study. The XBP1-EGFP sensor, originally developed by Sone et al(Sone et al., 2013)., is a widely used reporter for monitoring ER stress through Xbp1 splicing in real time(Du et al., 2021; Wang et al., 2014). This construct is designed to indicate the splicing status of Xbp1 mRNA and does not affect Xbp1-mediated transcriptional responses.*

*The XBP1-EGFP construct fuses the full-length Xbp1 sequence with enhanced green fluorescent protein (EGFP) (see image A below). In its unspliced form (XBP1u), the protein is cytoplasmic. Upon IRE1-mediated splicing of Xbp1 mRNA, the spliced form (XBP1s) translocates to the nucleus, allowing nuclear localization of XBP1-EGFP to serve as a direct readout of Xbp1 splicing activity (Fig.4, C to J). Importantly, under non-stressed conditions, the reporter only generates the unspliced form, XBP1u, and does not mimic the overexpression of the active spliced form (XBP1s). This feature differentiates it from constructs like UAS-Xbp1spliced, which are explicitly designed for overexpression studies(Wang et al., 2014).*

*We acknowledge the reviewer’s suggestion to assess the splicing of the native Xbp1 transcript to avoid potential artifacts associated with the reporter construct. To address this concern, we conducted RT-qPCR experiments to directly detect the spliced form of Xbp1, and the results showed no significant difference in xbp1s expression between the XBP1-EGFP system and the control group (see image B below). These findings are consistent with our observations using the XBP1-EGFP reporter, which does not translocate to the nucleus under non-stress conditions. Therefore, in this study, we primarily use XBP1-EGFP as a reporter line to indicate ER stress activation. These findings have been incorporated into the revised manuscript, and we have provided further clarification on the use and interpretation of the XBP1-EGFP system.*

|  |  |
| --- | --- |
| A. *The mechanism for the biosynthesis of XBP1 stress indicator (Sone et al., 2013)* | *B. RT-qPCR Analysis of Xbp1s Expression Levels* |

*(Fig.4, C to J)*

2. Regarding the function of Xbp1: It is surprising and inconsistent with the other results presented that Xbp1 knockdown improves proliferation in cells with a loss-of-function of WRNexo. As WRNexo is required and sufficient to induce proliferation and it presumably activates Xbp1, the converse would be expected, that WRNexo over-expression requires Xbp1 to induce proliferation.

*Response:*

*Thank you for the reviewer's insightful comments. We apologize for not clearly explaining the complex interplay between WRNexo, Xbp1, and Hsc70-3/BiP, which may have led to misunderstanding. Below, we clarify the mechanism underlying our findings.*

*Xbp1 is a central regulator in the IRE1 branch of UPR^ER^. Its spliced form (Xbp1s) promotes ER homeostasis by upregulating genes involved in protein folding and quality control, including Hsc70- 3/Bip. This allows UPR^ER^ activation to alleviate ER stress under normal conditions (see image below). Knockdown of Xbp1 (Xbp1 RNAi) impairs this protective role, leading to the accumulation of misfolded proteins, elevated ER stress, and activation of ROS-JNK signaling, which drives ISC proliferation. A similar effect is observed with Hrd1 RNAi, as it disrupts ER-associated degradation (ERAD) and increases ER stress(Wang et al., 2014) (see image below).*

*WRNexo overexpression disrupts ER homeostasis by binding to Hsc70-3/Bip and inhibiting its chaperone function. This inhibition activates ER stress, as evidenced by Xbp1 splicing and the nuclear localization of XBP1-EGFP. Notably, the activation of Xbp1 splicing is a passive response to ER stress. However, while Xbp1 splicing occurs, the downstream stress-mitigating effects that rely on functional Hsc70-3 are not fully realized. As a result, unresolved ER stress leads to pathological ROS-JNK activation and ISC hyper-proliferation (Fig.6Y). In this study, we primarily use XBP1-EGFP as a reporter line to indicate ER stress activation. In WRNexo RNAi flies failed to exhibit ISC proliferation after injury. However, knockdown of downstream regulators such as Xbp1 or Hrd1 increases ER stress and restores pathways like ROS-JNK signaling, thereby rescuing ISC proliferation in this background (Fig.4, K to Q). This suggests that regulating ER stress can compensate for the WRN deficiency phenotype.* *These findings illustrate that WRNexo functions as an upstream regulator of ER stress, and its interaction with Hsc70-3 is crucial for modulating UPR^ER^ signaling and ISC proliferation.*

*We have revised the manuscript to explicitly clarify these distinctions and the genetic interactions between WRNexo, Xbp1, and Hsc70-3/BiP. Thank you again for highlighting this important point, which has helped refine the clarity and depth of our work.*

Major branches of UPR^ER^ signaling pathways in *Drosophila*.

*(Figure1 B in Wang et al. 2014)*

*(Figure2 A and B in Wang et al. 2014)*

*(Fig.6Y)*

*(Fig.4, K to Q)*

3. Regarding Xbp1 and aging: the authors argue for a pro-aging role of WRNexo but this is somewhat inconsistent with the induction of Xbp1 in ISCs being able to promote organismal longevity and reduce the hyperplasia (e.g. Wang et al PLoS Genetics 2015; Li et al iScience 2024).

*Response:*

*Thank you for raising this important question. The ability of Xbp1 activation in ISCs to promote organismal longevity and reduce hyperplasia, as highlighted in studies(Li et al., 2024; Wang et al., 2014), is well-supported and aligns with its role as a key mediator of UPR^ER^. Xbp1 alleviates ER stress by driving the expression of stress-response genes, including Hsc70-3/BiP, which mitigates the accumulation of misfolded proteins and restores ER homeostasis. However, this protective effect relies on the proper functionality of Hsc70-3/BiP. Our findings suggest that WRNexo disrupts this pathway during aging. WRNexo binds to Hsc70-3/BiP, inhibiting its chaperone activity and impairing its ability to alleviate ER stress. Notably, the activation of Xbp1 splicing is a passive response to ER stress. And Hsc70-3/BiP function inhibition creates a scenario where ER stress accumulates, and although Xbp1 splicing indicates UPR^ER^ activation, the downstream effects that depend on Hsc70-3 are not realized. Instead, unresolved ER stress exacerbates ROS-JNK signaling, which promotes maladaptive ISC hyper-proliferation and intestinal dysplasia.*

*Notably, our experimental results demonstrate that reducing WRNexo expression during aging reduces ER stress, limits hyperplasia, and promotes lifespan extension (Fig.6, A to F). These findings are consistent with prior studies showing that alleviating ER stress enhances longevity and decreases ISC hyperplasia during aging. Together, these results underscore the critical role of WRNexo in regulating ER stress and its downstream consequences.*

*To clarify this point, we have revised the manuscript to emphasize the interplay between WRNexo, Hsc70-3/BiP, and Xbp1, and to explain how WRNexo-induced dysregulation of these pathways impacts intestinal homeostasis during aging. We appreciate the reviewer’s feedback, which has helped us refine our understanding and improve the clarity of our work.*

*(Fig.6, A to F)*

4. Bip is not only transcriptionally induced by Xbp1, it also regulates Xbp1 splicing (e.g. Kimata et al JCB 2004). This should be made clear throughout the text.

*Response:*

*Thank you for the reviewer’s valuable comment. We agree that BiP not only as a target of Xbp1’s transcriptional activation but also as a key regulator of Xbp1 splicing. Previous studies have shown that BiP, as an ER-resident chaperone, is transcriptionally induced by Xbp1s. In turn, BiP regulates Xbp1 splicing by modulating the activity of IRE1, a key sensor of ER stress, thereby contributing to the dynamic regulation of the unfolded protein response (UPR^ER^)(Kimata et al., 2004). To address this, we have revised the manuscript to include a more detailed explanation of BiP’s role in the regulation of Xbp1 throughout the text. Additionally, we have cited relevant literature (e.g., Kimata et al., 2004) to support these observations and ensure clarity. We appreciate the reviewer’s suggestion, which has helped improve the accuracy and comprehensiveness of our manuscript.*

Additionally, I would expect the authors to address they key aspects of the following concerns:

5. The authors present WRNexo as driving aging. They do show that it is required and sufficient for age-related hyperplasia, however, would it not be more appropriate to test if over-expression or knockdown of WRNexo in adult ISC is able to modulate lifespan in a way the authors would expect?

*Response:*

*We greatly appreciate the reviewer’s insightful suggestion regarding the lifespan experiments to further elucidate the role of WRNexo in regulating aging. In response to this suggestion, we have conducted additional lifespan experiments. Specifically, we overexpressed WRNexo in intestinal stem cells (ISCs) of young flies (7 days old) using the esg^ts^ with UAS-LacZ as the control. Our results show that overexpression of WRNexo in young ISCs significantly shortens the lifespan. Conversely, when WRNexo was knocked down in middle-aged flies (30 days old, maintained at 18°C until induction), we observed a marked extension of lifespan compared to the control group (Fig.6, E and F). These findings further substantiate our conclusion that WRNexo is a critical driver of age-related phenotypes, including aging-related hyperplasia and lifespan regulation. The details of these experiments and their results have been included in the revised manuscript.*

*(Fig.6, E and F)*

6. It does not appear that any of the fly lines used were backcrossed into a common background. Such backcrossing is essential when looking at aging or age-related phenotypes (e.g. lifespan, pH3+ accumulation in old flies etc.) in the fruit fly as differences in background could introduce false positives (see Partridge and Gems Nature 2007). The key experiments will have to be repeated with backcrossed lines.

*Response:*

*Thank you for the reviewer's insightful comment. We apologize for not providing sufficient details regarding this aspect in the original manuscript. As our laboratory focuses on aging research in Drosophila, we routinely backcross all fly lines used in aging-related experiments into the corresponding control strain for at least six generations. For this study,* *sibling populations were derived from crosses with w^1118^ to ensure genetic consistency. We have now included a detailed description of the backcrossing procedure in the revised manuscript under the Methods section. Thank you again for pointing out this important detail.*

7. In several figures, "control" is not clearly defined. It should be defined in the caption (driver alone, or a control RNAi, or simply wild-type flies?).

*Response:*

*Thank you for your helpful suggestion. We understand the importance of clearly defining the control group. To address this, we have now specified the type of control used in each figure caption, whether it refers to driver alone, control RNAi, or wild-type flies. These clarifications are now included in the figure legends. Additionally, where appropriate, we have added further control groups to ensure more robust comparisons.*

8. When analyzing data obtained by signal quantification from confocal images, I think it would be important to account for the fact that signal from different cells within one individual will be correlated by using e.g. mixed effects linear models.

*Response:*

*Thank you for the reviewer’s insightful comment. We agree that signal quantification from confocal images involves hierarchical data, as measurements from cells within the same individual are likely to be correlated. To address this, we carefully controlled all experimental steps, including dissection, staining, and imaging, ensuring that both experimental and control groups were processed under identical parameters.*

*Moreover, the Drosophila populations used in our study are sibling populations, which reduces genetic variability between individuals and minimizes differences unrelated to the experimental conditions. Additionally, we included a large sample size per group to account for potential between-individual variation, an approach widely recognized and applied in Drosophila studies(Du et al., 2021; Guo et al., 2024; Zhang et al., 2023).*

Rev. 3:

The authors present a study investigating the function of WRN in regulating ISC proliferation in response to aging. The further findings have significant implications that WRN can bind to Hsc70-3/Bip and regulate UPR. However, addressing the major concerns will strengthen the manuscript. To see specific comments below.

*Response:*

*We appreciate that the reviewer gives positive comments of our study.* *In order to address the issues mentioned by the reviewer, we have provided a detailed explanation of the reviewer’s concerns and outlined our experimental results in the current revised version.*

Major concerns:

1. In Figure 2C, the flies used are stated to be 30-day-old, whereas in Figures 2A, 2D, and 2E, the flies used are mentioned to be 45-day-old. To ensure accurate comparisons, it is recommended to use the same age groups for all figures.

*Response:*

*Thank you for pointing out the inconsistency in the age groups used in Figure 2. We sincerely apologize for this oversight and have addressed it by standardizing the age of aging flies to 40 days (maintained at 25°C) across all relevant experiments in Figure 2. To ensure accuracy and comparability, we repeated the ARM staining and gut length measurements using 40-day-old flies. The updated results are consistent with our previous findings. Specifically, epithelial cells in the WRNexo-null midguts of 40-day-old flies were more sparsely distributed compared to wild-type controls (Fig.2, B and C)*, *and the midgut length was significantly shorter in WRNexo-null flies compared to wild-type flies (Fig.2, A, D, and E). These adjustments and updated data have been incorporated into the revised manuscript and we are grateful for the reviewer for their valuable feedback.*

*(Fig.2, A to E)*

2. Figures 2L and 2M lack a control group for comparison.

*Response:*

*Thank you for your valuable suggestion. We apologize for the omission of a control group in Figures 2L and 2M. In the revised manuscript, we have added data for wild-type flies without PQ treatment, showing both GFP+ and pH3+ cells (Fig.2, A to E, and Supplemental Fig. S2. F to J). These additions provide a clear baseline for comparison and strengthen the interpretation of our results. We appreciate the reviewer’s feedback again.*

*(Fig.2, N to R)*

*(Supplemental Fig. S2. F to J)*

3. For Fig. 3, Q to T. the expression of WRNexo cDNA by esg-Gal4 rescued the proliferation defect of ISCs in WRNexo-null midguts after injury, but there are no Figs to show proliferation defect in WRNexo-null midguts.

*Response:*

*Thank you for highlighting this point. We apologize for not including representative pH3 images to illustrate the proliferation defect in WRNexo-null midguts. To address this concern, we have now included representative gut pH3 staining images for three groups in the revised manuscript (Fig.3, Q to T, and Supplemental Fig. S3. F to J). These images demonstrate that WRNexo-null midguts exhibit a clear proliferation defect following injury. Furthermore, overexpression of WRNexo cDNA driven by esg-Gal4 effectively rescues this proliferation defect. The data and images have been added to the revised manuscript, and we appreciate the reviewer’s suggestion to improve the clarity of our results.*

*(Fig.3, Q to T)*

*(Supplemental Fig. S3. F to J)*

4. The authors suggested that WRNexo contributes to the response to ER stress, therefore it should show that the ER stress can be rescued in response to TM treatment when WRNexo is blocked.

*Response:*

*Thank you for the reviewer’s insightful comment. We apologize if our original description was unclear and led to any misunderstanding. Our study found that WRNexo regulates ER stress through its interaction with Hsc70-3, thereby modulating ISC proliferation during gut regeneration and aging. The XBP1-EGFP line is a widely used reporter for monitoring ER stress(Du et al., 2021; Wang et al., 2014). Under ER stress, the spliced form (XBP1s) translocates to the nucleus, resulting in the nuclear localization of XBP1-EGFP(Sone et al., 2013). The observed reduction in XBP1-EGFP nuclear localization under TM treatment in WRN RNAi flies can be explained by the interplay between TM-induced ER stress and WRN’s role in modulating this stress.*

*Tunicamycin (TM) induces ER stress by inhibiting N-linked glycosylation, leading to the accumulation of misfolded proteins in the ER(Lin et al., 2021). When WRN is knocked down, the function of Hsc70-3 is disinhibited, allowing it to bind to misfolded proteins. This alleviates TM-induced ER stress, leading to decreased Xbp1 splicing and reduced XBP1-EGFP nuclear localization (Fig.4, G to J).*

*(Fig.4, G to J)*

5. The authors hypothesize that the overexpressed WRNexo interacts with Hsc70-3, inhibiting its function and subsequently inducing Xbp1 splicing. However, to further support this hypothesis, additional experiments should be conducted.

*Response:*

*Thank you for the reviewer's valuable suggestion. We appreciate the importance of providing further evidence for the functional interaction between WRNexo and Hsc70-3/Bip. Through mass spectrometry (MS) (IP-MS) analyses and AlphaFold prediction, we identified Hsc70-3/Bip as a binding partner of WRNexo. This interaction was further validated by immunoprecipitation and co-immunoprecipitation analyses, confirming that WRNexo indeed binds to Hsc70-3/Bip (Fig. 5, A to E, and Supplemental Fig. S4C). To explore the functional significance of this interaction, we conducted genetic experiments to assess its role in UPR^ER^ signaling. Specifically, knockdown of Hsc70-3/Bip mimicked the effect of WRNexo overexpression by inducing Xbp1 splicing, as indicated by the nuclear localization of XBP1-EGFP. Conversely, co-expression of Hsc70-3/Bip and WRNexo reduced this phenotype, suggesting that WRNexo modulates downstream UPR^ER^ signaling through its interaction with Hsc70-3/Bip (Fig.5, F to K).*

*These findings highlight the functional relevance of the WRNexo- Hsc70-3/Bip interaction in regulating UPR^ER^ signaling. We have included these results and their implications in the revised manuscript, and we thank the reviewer for their feedback, which has helped us refine our study.*

*(Fig.5, A to E)*

*(Supplemental Fig. S4C)*

*(Fig.5, F to K)*

6. In the last part of the result, the expression of WRNexo can repress DNA damage, however, the induction of DNA damage corresponded to an upregulation of WRNexo. The authors should provide explanations for supporting the findings.

*Response:*

*Thank you for highlighting this point. The purpose of this figure is to explore the age-related increase in WRNexo expression. Our findings suggest that WRNexo upregulation is a cellular response to DNA damage, where its exonuclease activity plays a critical role in processing DNA lesions and supporting repair pathways, aligning with its established role in maintaining DNA stability(Poot et al., 2004; Zhang et al., 2015). This upregulation is initially protective, helping to mitigate DNA damage and restore homeostasis. However, during aging, as DNA damage accumulates beyond repair capacity, WRNexo levels remain persistently elevated in an attempt to address the damage. This prolonged response appears insufficient to fully resolve the damage and may contribute to stress responses, such as ER stress, which are characteristic of aging-related phenotypes. To clarify this mechanism, we have expanded the discussion in the revised manuscript, providing a clearer interpretation of WRNexo’s role in both DNA repair and aging-related changes.*

Minor concerns:

1. The conclusion that "these data indicate that the depletion of WRNexo led to the loss of gut homeostasis upon aging in Drosophila" should be more specific.

*Response:*

*Thank you for your constructive feedback. We agree that the original conclusion could be made more specific to better reflect the findings. In the revised manuscript, we have refined the statement to clearly articulate the role of WRNexo depletion in aging-related gut homeostasis loss. The updated conclusion now reads: "These data indicate that the depletion of WRNexo in Drosophila leads to impaired gut homeostasis during aging, characterized by shortened gut length, dysregulated ISC proliferation, disrupted epithelial integrity, and* *shorter lifespan."*

2. "Following PQ treatment, ROS levels in esg+ cells of flies with esgts-Gal4-driven WRNexo RNAi expression were significantly lower than in the controls. (Fig. 6, I to K)". The authors should do more work for the similar grammar problems.

*Response:*

*Thank you for pointing out this grammatical issue. We have revised the sentence for clarity and consistency. The updated version reads:*

*"* *ROS levels in esg^+^ cells were significantly lower in WRNexo RNAi flies than in controls after PQ treatment (Fig. 6, P to R)." We also thoroughly reviewed the manuscript, corrected similar issues, and had it polished by a professional editing service to ensure the language is clear and professional. Thank you for your helpful feedback.*

*A certification from MogoEdit.com*

**References:**

Du, G., Liu, Z., Yu, Z., Zhuo, Z., Zhu, Y., Zhou, J., Li, Y., and Chen, H. (2021). Taurine represses age-associated gut hyperplasia in Drosophila via counteracting endoplasmic reticulum stress. Aging cell 20, e13319.

Fang, E.F., Hou, Y., Lautrup, S., Jensen, M.B., Yang, B., SenGupta, T., Caponio, D., Khezri, R., Demarest, T.G., Aman, Y., et al. (2019). NAD(+) augmentation restores mitophagy and limits accelerated aging in Werner syndrome. Nature communications 10, 5284.

Guo, X., Zhou, J., La, Y., Liu, X., Yuan, Y., Ye, J., Zhang, Z., Chen, H., Ma, Y., Zhong, Z., et al. (2024). Very long-chain fatty acids control peroxisome dynamics via a feedback loop in intestinal stem cells during gut regeneration. Developmental cell 59, 3008-3024.e3008.

Kamath-Loeb, A.S., Zavala-van Rankin, D.G., Flores-Morales, J., Emond, M.J., Sidorova, J.M., Carnevale, A., Cárdenas-Cortés, M.D., Norwood, T.H., Monnat, R.J., Loeb, L.A., et al. (2017). Homozygosity for the WRN Helicase-Inactivating Variant, R834C, does not confer a Werner syndrome clinical phenotype. Scientific reports 7, 44081.

Kimata, Y., Oikawa, D., Shimizu, Y., Ishiwata-Kimata, Y., and Kohno, K. (2004). A role for BiP as an adjustor for the endoplasmic reticulum stress-sensing protein Ire1. The Journal of cell biology 167, 445-456.

Kudlow, B.A., Kennedy, B.K., and Monnat, R.J., Jr. (2007). Werner and Hutchinson-Gilford progeria syndromes: mechanistic basis of human progeroid diseases. Nature reviews Molecular cell biology 8, 394-404.

Lautrup, S., Caponio, D., Cheung, H.H., Piccoli, C., Stevnsner, T., Chan, W.Y., and Fang, E.F. (2019). Studying Werner syndrome to elucidate mechanisms and therapeutics of human aging and age-related diseases. Biogerontology 20, 255-269.

Li, M., Shou, H., Martínez Corrales, G., Svermova, T., Franco, A.V., and Alic, N. (2024). Xbp1 targets canonical UPR(ER) and non-canonical pathways in separate tissues to promote longevity. iScience 27, 109962.

Lin, C.C., Ding, C.C., Sun, T., Wu, J., Chen, K.Y., Zhou, P., and Chi, J.T. (2021). The regulation of ferroptosis by MESH1 through the activation of the integrative stress response. Cell death & disease 12, 727.

Poot, M., Jin, X., Hill, J.P., Gollahon, K.A., and Rabinovitch, P.S. (2004). Distinct functions for WRN and TP53 in a shared pathway of cellular response to 1-beta-D-arabinofuranosylcytosine and bleomycin. Experimental cell research 296, 327-336.

Poot, M., Yom, J.S., Whang, S.H., Kato, J.T., Gollahon, K.A., and Rabinovitch, P.S. (2001). Werner syndrome cells are sensitive to DNA cross-linking drugs. FASEB journal : official publication of the Federation of American Societies for Experimental Biology 15, 1224-1226.

Saunders, R.D., Boubriak, I., Clancy, D.J., and Cox, L.S. (2008). Identification and characterization of a Drosophila ortholog of WRN exonuclease that is required to maintain genome integrity. Aging cell 7, 418-425.

Shamanna, R.A., Croteau, D.L., Lee, J.H., and Bohr, V.A. (2017). Recent Advances in Understanding Werner Syndrome. F1000Research 6, 1779.

Sone, M., Zeng, X., Larese, J., and Ryoo, H.D. (2013). A modified UPR stress sensing system reveals a novel tissue distribution of IRE1/XBP1 activity during normal Drosophila development. Cell stress & chaperones 18, 307-319.

Wang, L., Zeng, X., Ryoo, H.D., and Jasper, H. (2014). Integration of UPRER and oxidative stress signaling in the control of intestinal stem cell proliferation. PLoS genetics 10, e1004568.

Zhang, Q., Deng, K., Liu, M., Yang, S., Xu, W., Feng, T., Jie, M., Liu, Z., Sheng, X., Chen, H., et al. (2023). Phase separation of BuGZ regulates gut regeneration and aging through interaction with m(6)A regulators. Nature communications 14, 6700.

Zhang, W., Li, J., Suzuki, K., Qu, J., Wang, P., Zhou, J., Liu, X., Ren, R., Xu, X., Ocampo, A., et al. (2015). Aging stem cells. A Werner syndrome stem cell model unveils heterochromatin alterations as a driver of human aging. Science (New York, NY) 348, 1160-1163.
