# Supplementary material for: Werner syndrome exonuclease promotes gut regeneration and causes age-associated gut hyperplasia in Drosophila
Source: PLoS Biol. 2025 Apr 22;23(4):e3003121. doi: 10.1371/journal.pbio.3003121 (PMC12013949; doi:10.1371/journal.pbio.3003121)
Supplement: Attachment — Submitted filename: Response Letter to Reviewers.docx [file pbio.3003121.s026.docx]

Reviewer comments:

Rev. 1: Vilhelm Bohr - note that his reviewer has signed his review.

I think they have adequately responded to the reviewers concerns and that the MS should be acceptable.

*Response:*

*We appreciate your valuable input on our paper and are grateful for your recognition that we have adequately addressed the reviewers' concerns.*

Rev. 2:

I thank the authors for taking my comments on board. I think most have been addressed sufficiently well but there are two where I would appreciate the authors make small additional changes in the text.

*Response:*

*Thank you for your constructive feedback and for recognizing the improvements we have made in our manuscript. We have addressed the issues you raised regarding Xbp1 GFP reporter and the Drosophila backcrossing, providing further clarification and revisions. We trust that these adjustments resolve your concerns.*

Regarding my original comment (1) about the Xbp1 GFP reporter, it is clear even from the diagram provided by the authors in the rebuttal that this construct is reporting the splicing of Xbp1 and not simply its nuclear localisation. It would be good if the authors would make this clear on page 13. The qPCR shown in the rebuttal should also be shown in the manuscript (e.g. in a supplementary figure).

*Response:*

*We greatly appreciate the reviewer’s insightful suggestion. (1) As per the reviewer's comment, we have clarified on page 13 that: "The XBP1-EGFP reporter line is widely used to indicate Xbp1 splicing, with the nuclear translocation of Xbp1s serving as a visual marker of this splicing and ER-stress activation." (2) The qPCR results have been added to Supplemental Fig. S4C, and we have updated the manuscript on page 13 to describe this result.*

*Once again, we thank the reviewer for the valuable feedback, which has further improved the manuscript.*

Regarding my comment (6) about backcrossing: thank you for clarifying. However, I find that the information in the methods is still not sufficient. Please explicitly note which lines were backcrossed or write that all lines used in the manuscript were backcrossed, and provide information about how the unmarked mutants/constructs were backcrossed (I believe the Crispr mutants were not marked, apologies if I have misunderstood).

*Response:*

*We sincerely apologize for not providing a clear explanation of the backcrossing procedure in the methods section. To clarify, all lines used in the manuscript* *were backcrossed into the w^1118^ background for six generations, and sibling populations were derived from the crosses.*

*The unmarked fly lines* *(such as WRNexo-null mutants generated by the CRISPR/Cas9 system)* *were genotyped using non-lethal PCR genotyping with adult wings, as previously described (Carvalho et al., 2009; Chen et al., 2021).* *In summary, a pair of adult wings from individual Drosophila was collected and placed in PCR tubes. Next, 20 μL of PCR mix containing Platinum SuperFi DNA Polymerase (Invitrogen^TM^; Catalog #12351010) and amplification primers was added. The PCR products were subsequently analyzed using electrophoresis on a 1% agarose gel containing ethidium bromide.* *Only flies with the correct genotype were selected for the next round of backcrossing.* *The final offspring, which successfully passed the backcrossing process, were used for experimentation.*

*We have now included this detailed information in the methods section to address the concerns raised by the reviewer. Thank you again for your valuable feedback, which has greatly helped improve the clarity of our manuscript.*

Additional minor suggested changes:

Page 2, end of the abstract: "elevated WNRexo-mediated mechanism" - the expression is a bit clumsy - please change for clarity.

*Response:*

*Thank you for your constructive feedback. We apologize for the previous lack of clarity in our expression. We have revised the sentence as follows: "This study reveals how elevated WRNexo contributes to stem cell aging, providing new insights into organ aging and the pathogenesis of age-related diseases, such as colon cancer." on Page 2.*

*We believe this revision more clearly conveys the intended meaning. Thank you again for your valuable suggestion.*

Page 6, start of the results: "contrary to our prediction" - please explain the prediction and reasoning for clarity.

*Response:*

*Thank you for your insightful comment. We have clarified our reasoning in the revised manuscript.* *Given that WRN mutations cause Werner syndrome, a condition characterized by premature aging, one might expect WRN expression to decrease during natural aging. However, our findings revealed the opposite trend, with WRN levels increasing in aging colon crypts. To ensure clarity, we have revised the text on page 6 to explicitly explain this rationale.*

*We appreciate the reviewer’s suggestion, which has helped us improve the manuscript.*

Page 11, section title: "via regulate" - by regulating?

*Response:*

*Thank you for pointing out this issue. We have corrected the phrasing to "by regulating" as suggested on Page 11. We appreciate your attention to detail.*

Page 12: Hsp70Ba, Hsp70Bb, Hsp68, Hsp67Ba and Hsp67Bc are referred to as UPR-ER genes - please provide a reference for this. If they are not UPR-ER genes (as I belive they are not ER chaperones), it may be advisable to refer to them as "involved in proteostasis" or something similarly broad.

*Response:*

*Thank you for your insightful suggestion. We acknowledge that these genes are not classical UPR^ER^ genes but are involved in proteostasis. To ensure accuracy, we have revised the text on page 12 to refer to them as "genes involved in proteostasis" instead of "UPR^ER^-associated genes".*

Page 14, top of the page: "Xbp1 encodes a … signalling," - this is somewhat redundant.

*Response:*

*Thank you for your suggestion. To avoid redundancy, we have removed the repeated description of Xbp1 on page 14.*

Rev. 3: Aiguo Tian - note that this reviewer has signed his review.

My concerns have been addressed.

*Response:*

*Thank you for your feedback. We are glad to hear that your concerns have been addressed. We appreciate your time and consideration.*

**References:**

Carvalho, G.B., Ja, W.W., and Benzer, S. (2009). Non-lethal PCR genotyping of single Drosophila. BioTechniques 46, 312-314.

Chen, S., Lyanguzova, M., Kaufhold, R., Plevock Haase, K.M., Lee, H., Arnaoutov, A., and Dasso, M. (2021). Association of RanGAP to nuclear pore complex component, RanBP2/Nup358, is required for pupal development in Drosophila. Cell reports 37, 110151.
